# Genome Reduction and Microbe-Host Interactions Drive Adaptation of a Sulfur-Oxidizing Bacterium Associated with a Cold Seep Sponge

**DOI:** 10.1128/mSystems.00184-16

**Published:** 2017-03-21

**Authors:** Ren-Mao Tian, Weipeng Zhang, Lin Cai, Yue-Him Wong, Wei Ding, Pei-Yuan Qian

**Affiliations:** Division of Life Science, Hong Kong University of Science and Technology, Hong Kong; University of Chicago

**Keywords:** cold seep, genome analysis, sponge, symbiont

## Abstract

As the most ancient metazoan, sponges have established close relationships with particular microbial symbionts. However, the characteristics and physiology of thioautotrophic symbionts in deep-sea sponges are largely unknown. Using a tailored “differential coverage binning” method on 22-Gb metagenomic sequences, we recovered the nearly complete genome of a sulfur-oxidizing bacterium (SOB) that dominates the microbiota of the cold seep sponge *Suberites* sp. Phylogenetic analyses suggested that this bacterium (an unclassified gammaproteobacterium termed “Gsub”) may represent a new deep-sea SOB group. Microscopic observations suggest that Gsub is probably an extracellular symbiont. Gsub has complete sulfide oxidation and carbon fixation pathways, suggesting a chemoautotrophic lifestyle. Comparative genomics with other sponge-associated SOB and free-living SOB revealed significant genome reduction in Gsub, characterized by the loss of genes for carbohydrate metabolism, motility, DNA repair, and osmotic stress response. Intriguingly, this scenario of genome reduction is highly similar to those of the endosymbionts in deep-sea clams. However, Gsub has retained genes for phage defense and protein secretion, with the latter potentially playing a role in interactions with the sponge host. In addition, we recovered the genome of an ammonia-oxidizing archaeon (AOA), which may carry out ammonia oxidation and carbon fixation within the sponge body.

**IMPORTANCE** Sponges and their symbionts are important players in the biogeochemical cycles of marine environments. As a unique habitat within marine ecosystems, cold seeps have received considerable interest in recent years. This study explores the lifestyle of a new symbiotic SOB in a cold seep sponge. The results demonstrate that both this sponge symbiont and endosymbionts in deep-sea clams employ similar strategies of genome reduction. However, this bacterium has retained unique functions for immunity and defense. Thus, the functional features are determined by both the symbiotic relationship and host type. Moreover, analyses of the genome of an AOA suggest that microbes play different roles in biochemical cycles in the sponge body. Our findings provide new insights into invertebrate-associated bacteria in cold seep environments.

## Abstract (precis)

Sponges and their symbionts are important players in the biogeochemical cycles of marine environments. As a unique habitat within marine ecosystems, cold seeps have received considerable interest in recent years. This study explores the lifestyle of a new symbiotic SOB in a cold seep sponge. The results demonstrate that both this sponge symbiont and endosymbionts in deep-sea clams employ similar strategies of genome reduction. However, this bacterium has retained unique functions for immunity and defense. Thus, the functional features are determined by both the symbiotic relationship and host type. Moreover, analyses of the genome of an AOA suggest that microbes play different roles in biochemical cycles in the sponge body. Our findings provide new insights into invertebrate-associated bacteria in cold seep environments.

## INTRODUCTION

Arising 600 million years ago, sponges are probably the most ancient living metazoan (1), and they play important roles in the biogeochemical cycle in the benthic zone (2). Sponges can be found in a variety of habitats such as deep-sea cold seeps. The deep-sea seeps studied so far include the Shaban Deep (3), the Discovery Deep (4), the Atlantis II Deep (5), and the Kebrit Deep (6). The seeps are diverse, with varied fluid flow regimes that support ecosystems with different structures (7). Our recent work (8–10) has investigated microbial communities in Thuwal cold seeps II, a new cold seep system found in 2012 (11). The Thuwal cold seeps are located in the central Red Sea at a depth of 850 m. The seep water is enriched with metals at 10 times the concentration of normal deep-sea water, including aluminum (0.24 ppm), arsenic (0.34 ppm), copper (0.20 ppm), and iron (0.10 ppm) (11). The collected water had a characteristic foul odor, indicating a high hydrogen sulfide content, and the concentration of sulfate is 2.7 g/liter (12).

Sponges maintain a close relationship with many microbes (13, 14). The microorganisms in the sponge body can reach up to 40% of the sponge weight and are mainly distributed in the mesophyll layer (15). A variety of microorganisms inhabit sponges, including *Proteobacteria*, *Bacteroidetes*, *Cyanobacteria*, *Firmicutes*, *Chloroflexi*, and *Actinobacteria*, as revealed by 16S rRNA sequence data (16). Comparisons with microbial communities in the surrounding water have revealed the unique composition and phylogeny of sponge-associated microorganisms (17, 18). Microbial communities in symbiosis with six sponge species show functional equivalence and evolutionary convergence, suggesting the existence of core functions for sponge-associated microorganisms (19). Moreover, a metagenomic investigation by Thomas et al. (18) revealed potential metabolic interactions between bacteria and the sponge host, including vitamin production, nutrient transport, as well as redox sensing and response. These findings all point to interactions between microbes and the host sponge, contributing to the widely observed host specificity of these microbial groups. More-recent studies have employed genomics to explore the functional features of single microbes associated with sponges. Examples include a first-draft genome of “*Candidatus* Synechococcus spongiarum,” which inhabits the Red Sea sponge *Carteriospongia foliascens* (19); single-cell genomes of members of *Entotheonella*, which are widely distributed in sponges (12), and three additional draft genomes of “*Ca*. Synechococcus spongiarum,” each from a different clade (20). According to these genomic studies, sponge-associated microbes show functional dissimilarities to closely related free-living strains, for example, the unique chemical compounds produced by *Entotheonella* and gene deletion in “*Ca*. Synechococcus spongiarum.”

As potential autotrophic and carbon-providing symbionts, thioautotrophic microbes have attracted a large amount of research interest since the 1970s (21). Symbiotic bacteria in tube worms (22, 23), clams (24, 25), mussels (26), snails (27), and sponges (28) have been found to interact with their host intensively. These symbionts are mostly sulfur-oxidizing bacteria (SOB) that scavenge reduced sulfur from the hosts and provide vitamins required by the hosts (25, 26). In addition, the hosts acquire food from the bacterial symbionts and provide them with shelter. Genomic studies have investigated several sulfur-oxidizing symbionts from deep-sea invertebrates, including “*Candidatus* Ruthia magnifica” strain Cm in the clam *Calyptogena magnifica* (25), “*Candidatus* Vesicomyosocius okutanii” strain HA in the clam *Calyptogena okutanii* (26), a symbiont of the oligochaete *Olavius algarvensis* (29), a gammaproteobacterium in the snail *Crysomallon squamiferum* (27), and a gammaproteobacterium in the deep-sea glass sponge *Lophophysema eversa* (30). In* Lophophysema eversa*, the microbial community was dominated by an ammonia-oxidizing archaeon (AOA), a nitrite-oxidizing bacterium (NOB), and a SOB, all of which were autotrophs. In addition, an experimental study has demonstrated that the high demand for oxygen by chemoautotrophic symbionts could be a major factor precluding their endosymbiosis with cnidarians (31).

One important feature for a variety of endosymbionts is genome reduction. These microorganisms in hosts lack genes that are essential in other bacteria and retain only the most essential functions, such as sets of genes that serve the hosts (32). Genome reduction is a hallmark of the genomes of symbionts and is considered a strategy to reduce the cost of genome replication (33). It is also considered to result from coevolution between the endosymbiont and host (34), and it can be neutral or adaptive (35).

Although thioautotrophic symbionts of advanced marine animals, such as clams, have been studied widely (reviewed in reference 21), there is limited knowledge of the symbionts of sponges in deep-sea environments, especially in cold seeps. Given that sponges have no differentiated tissue (36) and that the microenvironment in sponge bodies is unlike other invertebrates (e.g., oxygen levels vary spatially) (37), it is interesting to explore the adaptation of symbionts in sponge bodies. Here, we have successfully recovered the nearly complete genome of a sponge-associated SOB from the sponge *Suberites* sp. (classified to the genus level based on its 18S rRNA gene, which shows 99% identity over 1,727 bp to the 18S rRNA gene of *Suberites* sp. strain 0M9H2772-G) collected at the Thuwal cold seeps II. Using comparative genomics, we have demonstrated unique ecological functions and adaptive mechanisms in this bacterium.

## RESULTS AND DISCUSSION

### Metagenome information and genome binning.

Two metagenome pairs (external/internal tissues and sponge cell-enriched/prokaryote cell-enriched samples; see Materials and Methods for the details) were sequenced using different platforms (HiSeq and MiSeq), to improve DNA assembly and microbial genome binning from the mixed eukaryotic and prokaryotic DNA sequences. In total, 4.7 and 4.4 Gbp of HiSeq2000 reads of the external and internal tissue metagenome, respectively, were obtained. After quality control and assembly, 27,109 contigs larger than 500 bp (total length, 102 Mbp) were generated. In parallel, 6.2 and 6.4 Gbp of the MiSeq (2 × 300-bp) reads of the sponge cell- and prokaryote cell-enriched samples, respectively, were obtained. After quality control, 5.6 and 5.3 Gbp of high-quality reads were retained and assembled with the contigs from the external and internal tissue metagenomes. The final assembly resulted in 41,405 contigs (a total length of 132 Mbp). The improved assembly was used in the subsequent genome binning process. The qualified HiSeq and MiSeq sequences and the final assembly are summarized in Table S1 in the supplemental material.

10.1128/mSystems.00184-16.7 TABLE S1  Information on the unassembled and assembled metagenomes. The statistics of Illumina HiSeq and MiSeq paired-end reads of the sponge samples, the HiSeq reads of the water sample after quality control using the NGS QC toolkit, and the statistics of assembled contigs (>500 bp) are shown. Download TABLE S1, PDF file, 0.1 MB. Copyright © 2017 Tian et al. 2017 Tian et al. This content is distributed under the terms of the Creative Commons Attribution 4.0 International license.

**FIG 1:**
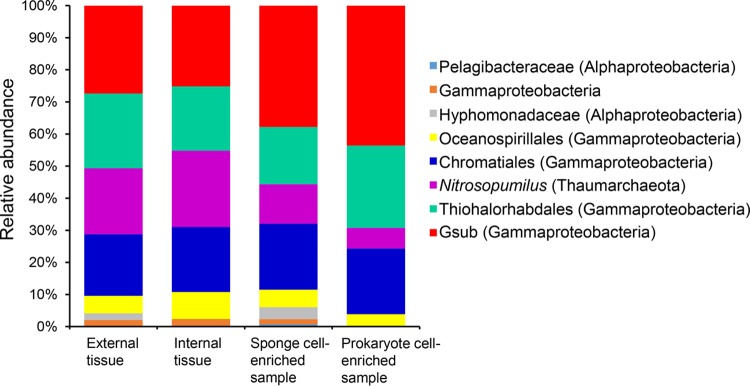
Microbial community composition of the samples, including external and internal tissues, sponge cell- and prokaryotic cell-enriched samples. The red block represented the Gsub bacterium in the present study. The sequencing coverages of the contigs where the 16S rRNA genes located were taken as the coverages of the 16S rRNA genes and were considered their relative abundance. The lowest assigned taxonomic levels are shown. The phylum names and the class names for *Proteobacteria* are shown in parentheses.

Analysis of the prokaryotic community composition of the sponge samples (Fig. 1) indicated that there were four dominant species, namely, an archaeon from the genus *Nitrosopumilus* and three gammaproteobacteria, one from the order *Thiohalorhabdales*, one from the order *Chromatiales*, and one from an unclassified order. *Thiohalorhabdales* were previously isolated from hypersaline lakes and considered to be SOB (38); in a recent study, the abundance of *Thiohalorhabdales* was correlated with the nitrite concentration, suggesting a potential role in nitrogen cycling (39). Members of the archaeal genus *Nitrosopumilus* are distributed in global oceans and perform the functions of nitrification and autotrophy (40). Members of *Chromatiales* are often obligately aerobic chemoheterotrophs, which can also oxidize sulfur (41). The unclassified gammaproteobacterium was a SOB, based on our subsequent phylogenetic and genomic analyses. Thus, the cold seep sponge-associated microbiome is dominated by autotrophic microbes, which may play important roles in the carbon, nitrogen, and sulfur cycles. Metagenomics of the water sample collected during the same cruise were also analyzed (details for the metagenome information are summarized in Table S1), revealing that the sponge-associated microbial community is less diverse than that of the water (Fig. S1).

10.1128/mSystems.00184-16.1 FIG S1  Microbial community composition of the surrounding water sample collected at the same time as sponge collection. The sequencing coverage of the contigs where the 16S rRNA genes located were taken as the coverage of the 16S rRNA genes and were considered their relative abundance. The lowest assigned taxonomic level according to Greengene is shown. Download FIG S1, PDF file, 0.1 MB. Copyright © 2017 Tian et al. 2017 Tian et al. This content is distributed under the terms of the Creative Commons Attribution 4.0 International license.

In the present study, we focus on the unclassified gammaproteobacterium and we will refer to it as “Gsub” hereafter. Recovery of the Gsub genome was conducted by comparing the sequencing coverage of the contigs in the external and internal tissue samples, as well as in the sponge and prokaryote cell-enriched samples. In addition to the sequencing coverage grouping, tetranucleotide frequency clustering was also applied to collect a purer genome (Fig. S2). Intriguingly, the two approaches recovered exactly the same contigs for the Gsub genome, indicating the accuracy of the binning method used in the present study. In addition, the similar relative abundance of Gsub in the external and internal samples suggested a strong association of this bacterium with the sponge. The bin of the Gsub genome contained 14 contigs (total length of 1,371,853 bp; total sequencing coverage of 745×) with a maximum length of 212 kbp and a mean length of 98 kbp. The genome completeness and potential contamination of the Gsub genome were estimated to be 98% and 0%, respectively, based on the identification of 243 *Proteobacteria*-specific single-copy marker genes using CheckM (42). The Pfam-based 139 conserved single-copy genes were also identified, showing that the Gsub genome contains no duplicated genes (Table S2). The high completeness and purity of the genome allow us to investigate the phylogeny and potential lifestyle of Gsub.

10.1128/mSystems.00184-16.2 FIG S2  Binning of the Gsub bacterium. (A to D) Binning by comparing the sequencing coverage of the contigs in the external and internal tissue samples. (A) Coverage of all contigs (the area indicates contig length, and the color indicates taxonomy at the phylum level; the axes are in log scale). (B) Contig group selected for the Gsub bacterium. Some additional contigs were included to test the clustering of tetranucleotide frequency (TNF) in panel D. (C) Correspondence analysis (CA) from CA1 to CA5. CA decomposes the chi-square statistic associated with the two-way table into orthogonal factors that maximize the separation between row and column scores. CA1 to CA5 are the 5 most important components of the CA analysis. (D) The CA clustering of CA1 and CA2, which further purifies the bacterium Gsub genome. (E to H) Binning of Gsub by comparing the sequencing coverage of the contigs in the sponge cell- and prokaryote cell-enriched samples. Download FIG S2, PDF file, 0.3 MB. Copyright © 2017 Tian et al. 2017 Tian et al. This content is distributed under the terms of the Creative Commons Attribution 4.0 International license.

10.1128/mSystems.00184-16.8 TABLE S2  Distribution of the 139 hidden Markov models (HMMs) of single-copy protein-coding genes for the Gsub bacterium and the reference bacteria. Download TABLE S2, PDF file, 0.2 MB. Copyright © 2017 Tian et al. 2017 Tian et al. This content is distributed under the terms of the Creative Commons Attribution 4.0 International license.

### Phylogeny of Gsub.

**FIG 2:**
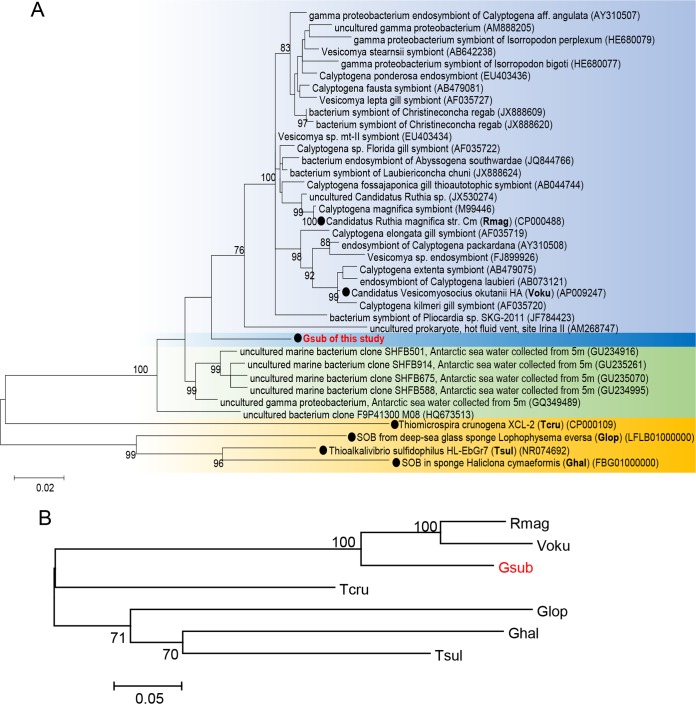
Maximum likelihood (ML) unrooted tree based on the full-length 16S rRNA genes (A) and 31 conserved single-copy genes (B) showing the phylogenetic position of the Gsub bacterium and its close relatives. GenBank accession numbers of the reference sequences are shown in parentheses, and the Gsub bacterium is found to be closely related to the cluster of endosymbionts of the deep-sea clam, with the highest 16S rRNA gene identity of 94.5% to “*Candidatus* Ruthia magnifica” strain Cm. The black circles indicate the reference genomes involved in the subsequent genomic comparison. Bootstrap values of >70% are shown. The length of the branch in the horizontal dimension indicates the amount of change in the evolutionary lineage. The bars at the bottom of panels A and B represent 0.02 and 0.05 nucleotide substitutions per position. aff., affiliated; str., strain.

Gsub shares the highest 16S rRNA gene identity (94.5%) with “*Candidatus* Ruthia magnifica” strain Cm, an intracellular SOB found in the deep-sea clam *Calyptogena magnifica* (25). The phylogenetic tree (Fig. 2A) shows that Gsub is close to a group of thioautotrophic endosymbionts of the deep-sea *Bivalvia* represented by “*Candidatus* Ruthia magnifica” Cm (25) and “*Candidatus* Vesicomyosocius okutanii” HA (24). Two free-living SOB and two sponge-associated SOB were also included in the phylogenetic tree as outgroups, and they showed an 84.8 to 87.0% 16S rRNA gene identity to Gsub. In addition to the 16S rRNA gene tree, 31 single-copy genes were used to determine the phylogenetic position of Gsub, and the result revealed a similar relationship between Gsub and the reference bacteria (Fig. 2B). The *soxB* genes are largely unique to SOB and have been targeted as a means of examining the diversity of marine SOB (43). Therefore, a tree based on the *soxB* gene sequences was constructed, demonstrating that *soxB* from Gsub has an 83.0 to 87.5% match with the closest sequences and forms an independent branch (Fig. S3). The phylogenetic analyses indicate that Gsub may represent a novel SOB group.

10.1128/mSystems.00184-16.3 FIG S3  ML tree based on the full-length SoxB proteins. The SoxB protein sequence in Gsub showed the highest identity (87.5%) to the thioautotrophic gill symbiont of* Bathymodiolus azoricus*. Bootstrap values of >70% are shown. Download FIG S3, PDF file, 0.1 MB. Copyright © 2017 Tian et al. 2017 Tian et al. This content is distributed under the terms of the Creative Commons Attribution 4.0 International license.

### Microscopic observations.

To further investigate the association between Gsub and the sponge, fluorescence in situ hybridization (FISH) experiments were performed using sponge sections with a Gsub-specific probe for the 16S rRNA gene, and the results found Gsub in extracellular positions in the sponge (Fig. S4). The Gsub bacteria are cocci with a diameter of ~1 µm, and they stained red with the specific probe. The negative control showed no signal with the negative probe.

10.1128/mSystems.00184-16.4 FIG S4  Fluorescence *in situ* hybridization (FISH) experiment on the sponge tissue using a Gsub-specific probe for the target sulfur-oxidizing bacterium (SOB). (A and B) Sponge cells (indicated by the arrow) and prokaryotic cells stained with DAPI which exhibit blue fluorescence (note that there were many prokaryotic cells with weak fluorescence). (C) Bacteria stained with the Cy3-labeled universal probe EUB338 (red fluorescence) corresponding to the field shown in panel A. (D) Target Gsub stained with Cy3-labeled Gsub-specific probe corresponding to the field shown in panel B. (E and F) Bright-field microscopy views of panels A and B, respectively. Download FIG S4, PDF file, 0.2 MB. Copyright © 2017 Tian et al. 2017 Tian et al. This content is distributed under the terms of the Creative Commons Attribution 4.0 International license.

### Chemoautotrophic metabolism.

The Gsub genome contains a complete set of genes involved in the reverse dissimilatory sulfate reduction pathway (*dsrA*, *dsrB*, *aprA*, *aprB*, and *sat*) and the sulfur oxidation (SOX) system (*soxABXYZ*), indicating that this bacterium is able to use both sulfide and thiosulfate as electron donors (Fig. S5A). The Gsub genome also contains a complete set of genes involved in the Calvin-Benson cycle for carbon fixation, including the *rbcS* and *rbcL* genes coding for ribulose-bisphosphate carboxylases (RubisCO) (Fig. S5B). The presence of pathways for sulfur oxidation and carbon fixation indicates a genetic capability for chemoautotrophic metabolism. In addition, different types of transporters were identified from the Gsub genome, including those for iron, zinc, and l-amino acid transport, suggesting that the symbiont may receive nutrients from the sponge host.

10.1128/mSystems.00184-16.5 FIG S5  Metabolic pathway reconstruction for the Gsub bacterium. (A) Kyoto Encyclopedia of Genes and Genomes (KEGG) pathway annotation showed that the Gsub bacterium contained a complete set of genes involved in reverse dissimilatory sulfate reduction (*dsrAB*, *aprAB*, and *sat*) and genes involved in the sulfur oxidation system (*soxABXYZ*, but not *soxCD*). (B) Calvin-Benson cycle. (C and D) Gsub lacks flagellum genes (C) and chemotaxis genes (D), which is consistent with the two thioautotrophic endosymbionts (Rmag and Voku) of vent clams. The two free-living SOB (Tsul and Tcru) have nearly complete sets of flagellum and chemotaxis genes, and the other genomes lack them. The seven units of the rectangles indicated Gsub, Rmag, Voku, Glop, Ghal, Tsul, and Tcru, respectively. Download FIG S5, PDF file, 0.1 MB. Copyright © 2017 Tian et al. 2017 Tian et al. This content is distributed under the terms of the Creative Commons Attribution 4.0 International license.

### Genome reduction of Gsub.

**FIG 3:**
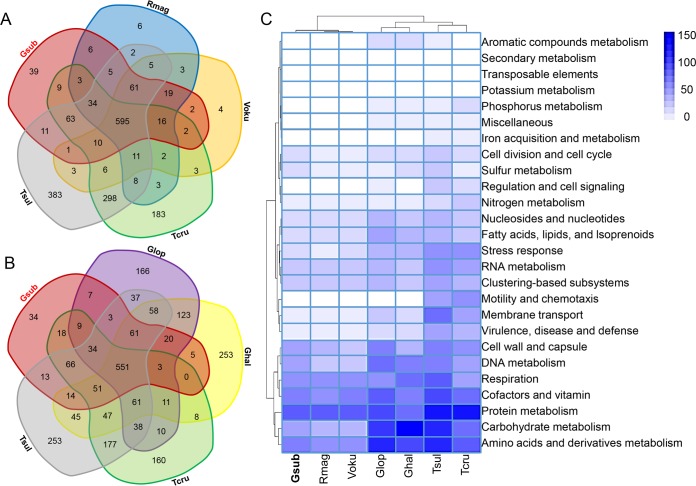
Comparison of functions between the Gsub bacterium and the reference genomes. (A) Venn diagram showing the distribution of Kyoto Encyclopedia of Genes and Genomes (KEGG) genes in the Gsub, Rmag, Voku, Tcru, and Tsul bacteria. (B) Venn diagram showing the distribution of KEGG genes in the Gsub, Glop, Ghal, Tcru, and Tsul bacteria. (C) Heatmap and clustering of the functional profile (first level of SEED subsystem) in the genomes of Gsub and reference bacteria. The genomes of Gsub, Rmag, and Voku contain lower numbers of genes for carbohydrate metabolism and respiration, cofactor synthesis, cell signaling and regulation, virulence factors, motility and chemotaxis, stress response, and membrane transport.

To gain better understanding of the functional profile of Gsub, we compared its genome to two of its nearest neighbor genomes: two endosymbiont SOB (“*Candidatus* Ruthia magnifica” strain Cm [Rmag] and “*Candidatus* Vesicomyosocius okutanii” strain HA [Voku]) from vent clams (24, 25); the comparison also included two potential sponge symbiont SOB (the SOB in the deep-sea glass sponge *Lophophysema* [Glop] and in the shallow water sponge *Haliclona* [Ghal]) (30, 44) and two free-living SOB (*Thioalkalivibrio sulfidophilus* HL-EbGr7 [Tsul] and *Thiomicrospira crunogena* XCL-2 [Tcru]) from shallow and deep water environments (Table 1). The overall genomic picture of Gsub was highly similar to that of the Rmag and Voku, which possess a much lower number of unique Kyoto Encyclopedia of Genes and Genomes (KEGG) genes (4 to 39) than the free-living bacteria (183 to 383) (Table 1 and Fig. 3A). The 39 unique KEGG genes for Gsub compared to Rmag, Voku, Tcru, and Tsul are listed in Table S3. In particular, the genes for nitrite reduction (*nirK*) and nitric oxide reduction (*norC*) are unique in Gsub, suggesting that Gsub uses different strategies of anaerobic respiration than the other four bacteria do. A second Venn diagram was drawn to show the genes in common between sponge-associated and free-living SOB (Fig. 3B; comparing Gsub with Glop, Ghal, Voku, and Tsul). This shows that 34 genes are unique to Gsub (Table S3). Again, *nirK* and *norC* were present only in Gsub. In particular, one of the 34 unique genes is *wbqP*, which encodes O-antigen biosynthesis protein, and O antigen is used by bacteria to avoid the induction of the host immune system to facilitate the establishment of the symbiosis relationship in plants (45). A heatmap of functional categories based on the SEED subsystem shows a high similarity of the genomic profiles of Gsub, Rmag, and Voku, and these three bacteria formed a distinct group from the other bacteria. These three genomes have lost a number of genes involved in carbohydrate metabolism, respiration, cofactor synthesis, cell signaling and regulation, motility and chemotaxis, stress response, and membrane transport (Fig. 3C).

10.1128/mSystems.00184-16.9 TABLE S3  The 39 unique KEGGs for Gsub compared with Rmag, Voku, Tcru, and Tsul, as revealed by the Venn diagram in Fig. 3A, and the 34 unique KEGGs for Gsub compared with Glop, Ghal, Tcru, and Tsul, as revealed by the Venn diagram in Fig. 3B. Download TABLE S3, PDF file, 0.1 MB. Copyright © 2017 Tian et al. 2017 Tian et al. This content is distributed under the terms of the Creative Commons Attribution 4.0 International license.

**TABLE 1:** Comparison of the genome features among the sulfur-oxidizing bacterium (SOB) in the cold seep sponge (Gsub) and reference genomes^a^

Genome feature	Gsub	Rmag	Voku	Glop	Ghal	Tsul	Tcru
Habitat	Deep-sea sponge	Deep-sea clam	Deep-sea clam	Deep-sea sponge	Shallow-water sponge	Shallow water free-living	Deep-sea free-living
Accession no.	JYIN01000000	CP000488	AP009247	LFLB01000000	JFBG01000000	CP001339	CP000109
Completeness (%)	98.00	94.16	94.19	94.51	99.56	99.89	99.39
Potential contamination (%)	0	0	0	2.44	1.01	0.34	0
Total length (Mbp)	1.4	1.2	1.0	2.7	3.5	3.5	2.4
No. of protein-coding genes	1,370	1,076	939	2,506	2,741	3,319	2,201
No. of tRNA genes	36	36	36	43	44	48	44
Reduced features							
No. of genes involved in:							
Monosaccharide metabolism	4	4	3	10	32	17	12
Sugar alcohol metabolism	2	2	2	17	21	8	3
Oligosaccharide metabolism	2	0	0	7	8	11	11
Motility and chemotaxis	4	4	3	6	9	65	76
Resistance to toxic compounds and heavy metals	14	14	14	17	23	57	55
Oxidative stress	14	11	13	22	20	27	17
Osmotic stress	0	0	0	10	14	5	9
DNA repair	31	21	16	39	39	46	36
Retained features							
No. of type II secretion system genes	11	0	0	0	8	12	12
No. of CRISPR sites	3	0	0	6	0	2	0
No. of CRISPR spacers	16	0	0	191	0	71	0
No. of CRISPR protein	2	0	0	14	4	5	0

a The reference genomes include two endosymbionts from vent clams, “*Candidatus* Ruthia magnifica” strain Cm (Rmag) and “*Candidatus* Vesicomyosocius okutanii” strain (Voku), two extracellular SOB in sponges, the SOB in the deep-sea glass sponge *Lophophysema* (Glop) and in the shallow water sponge *Haliclona* (Ghal), and two free-living relative SOB, *Thioalkalivibrio sulfidophilus* HL-EbGr7 (Tsul) from shallow water and *Thiomicrospira crunogena* XCL-2 (Tcru) from the deep sea.

Osmotic stress resistance genes for choline and betaine uptake could not be detected in Gsub, Rmag, or Voku, but there were an average of nine such genes in Glop, Ghal, Tsul, and Tcru (Tables 1 and 2). Statistics using Student’s *t* test indicate that the difference between the gene numbers in these two groups of microbes was significant (*P* < 0.05). The roles of glycine, betaine, and choline in osmoprotection have been demonstrated in a variety of microbes, such as members of *Rhizobium*, *Sinorhizobium*, *Mesorhizobium*, and *Agrobacterium* (46). In the cold seep environment, high osmolarity is probably an external stress for free-living microbes, because of the high concentration of metals. However, the loss of genes related to osmotic stress resistance in Gsub suggests that the microenvironments of host bodies is likely a stable environment for bacteria. This concept is consistent with previous findings for sponge symbionts: in the studies by Gao et al. (19) and Burgsdorf et al. (20), oxidative stress resistance genes were reduced in symbiotic members of the “*Candidatus* Synechococcus spongiarum” group, and the authors proposed that the barrier of the sponge body prevents sunlight from arriving at the cyanobacterial symbiont, avoiding oxidative damage.

**TABLE 2:** Comparison of detailed genomic features on the resistance to toxic compounds and heavy metals, oxidative stress, osmotic stress, and DNA repair in the bacterium Gsub genome and reference bacteria

Gene category and annotation^a^	No. of genes in the following bacterium with the indicated genes:						
	Gsub	Rmag	Voku	Glop	Ghal	Tsul	Tcru
Resistance to toxic compounds and heavy metals							
Copper homeostasis	2	3	3	3	4	5	7
Multidrug resistance efflux pumps	3	3	3	2	6	11	11
Cobalt-zinc-cadmium resistance	1	1	1	4	3	21	25
Resistance to fluoroquinolones	4	4	4	4	4	4	4
Copper tolerance	1	1	1	2	2	4	2
Zinc resistance	0	0	0	0	0	2	0
Multidrug resistance, tripartite systems	0	0	0	0	1	0	0
Beta-lactamase	0	0	0	1	1	2	2
Multidrug efflux pump (CmeABC operon)	0	0	0	0	0	0	2
Methicillin resistance	2	1	1	1	2	1	1
Arsenic resistance	1	1	1	0	0	7	1
Subtotal	14	14	14	17	23	57	55

Oxidative stress							
Rubrerythrin	4	1	2	4	3	6	2
Glutathione: biosynthesis and gamma-glutamyl cycle	3	2	3	5	4	3	5
Redox-dependent regulation of nucleus processes	0	0	1	3	4	4	2
Glutaredoxins	3	3	3	2	1	5	2
Glutathione							
Nonredox reactions	1	2	1	5	7	3	4
Redox cycle	3	3	3	3	1	6	2
Subtotal	14	11	13	22	20	27	17

Osmotic stress							
Betaine biosynthesis from glycine	0	0	0	0	0	2	0
Ectoine biosynthesis and regulation	0	0	0	0	0	0	5
Choline and betaine uptake	0	0	0	10	14	3	4
Subtotal	0	0	0	10	14	5	9

DNA repair							
MutL-MutS system	0	0	0	2	2	3	2
Bacterial DNA repair	10	7	4	16	15	18	12
RecFOR pathway	5	1	1	6	8	7	6
Base excision	7	6	6	7	6	8	5
UvrABC system	3	3	1	3	3	4	4
UvrD and related helicases	3	1	2	2	3	2	3
DinG and relatives	1	1	1	1	0	1	1
2-Phosphoglycolate salvage	2	2	1	2	2	3	3
Subtotal	31	21	16	39	39	46	36

a Genes were annotated using the SEED database.

In the DNA repair category, the MutL-MutS system was absent in Gsub, Rmag, and Voku, while present in Glop, Ghal, Tsul, and Tcru (Tables 1 and 2). The difference between the gene numbers in these two groups of microbes was significant (*P* < 0.05). The *mutL*, *mutS*, and *dcm* genes in bacteria participate in the repair of mismatches at 5-methylcytosine sites (47). In addition, genes encoding components of the RecFOR pathway, which is involved in DNA double-strand break repair (48), also had lower numbers in Gsub, Rmag, and Voku. Elimination of some of the genes encoding components of the DNA recombination and repair pathways has been observed for nearly every small genome (49). In the study of the sponge symbiont “*Candidatus* Synechococcus spongiarum” by Gao et al. (1,9), DNA repair enzymes, including the exonuclease Exo VII complex in the mismatch repair pathway, the exonuclease V complex (RecBCD) in the homologous recombination pathway, and ATP-dependent DNA ligase were missing. However, it is not clear how these microbes benefit by retaining fewer DNA repair genes.

With respect to carbohydrate metabolism, the Gsub genome has reduced genes for the metabolism of monosaccharides, oligosaccharides, sugar alcohols, and other carbohydrates compared to free-living relatives. Enzymes involved in polysaccharide synthesis, including starch synthase and UDP-glucose-6-dehydrogenase, were not detected in the Gsub genome. The sponge body is a microenvironment providing rich organic and inorganic nutrients (50, 51). The loss of some genes for carbohydrate biosynthesis implies that Gsub may be able to access these nutrients from the host, consistent with the above-mentioned analyses on transporters.

In terms of motility, Gsub lacked all the genes encoding bacterial flagellar components, including the basal body, motor, rings, hook, filament, and regulatory proteins, and the corresponding chemotaxis proteins (Table 1; Fig. S5C and S5D). In addition, no pilus genes that are involved in gliding motility could be detected in Gsub. This provides evidence for an immobile lifestyle in the sponge body. The reduced bacterial motility in Gsub implies that symbionts in the sponge body are sessile and do not need to move in response to chemical signals as either a response to stress or a strategy of nutrient acquisition.

### Immunity and virulence genes in Gsub.

Clustered regularly interspaced short palindromic repeats (CRISPRs) are responsible for phage-specific defense (52). Comparison of CRISPR-associated proteins and CRISPR sites differentiated Gsub from the two endosymbiotic bacteria Rmag and Voku: Gsub retained the CRISPR-associated proteins Cas1 and Cas2, which were absent in Rmag and Voku; three CRISPR sites containing 2, 4, and 10 spacers were found in the Gsub genome, but none were found in the Rmag and Voku genomes (Table 3). In the deep-sea glass sponge *Lophophysema eversa*, the three dominant species (three autotrophs) all were enriched in CRISPRs and associated proteins compared to their free-living relatives (53). Comparison of metagenomic data from sponge-associated microbial communities with planktonic communities in the surrounding water also showed the increased presence of functions of CRISPR in sponge-associated bacteria (17). Given the high water pumping rate of sponges, the bacteria in sponges are exposed to a high risk of phage infection, and this may explain the absence of CRISPR in the two clam symbiont genomes Rmag and Voku. Moreover, the absence of CRISPR in Rmag and Voku could also be attributed to their smaller genomes, consistent with the concept that the high genetic load of the phage resistance system could be too great for certain small genomes (20). The presence of CRISPR in the Gsub genome, combined with the results of previous studies (17, 53), implies that phage defense is of ecological significance in the adaptation of sponge-associated bacteria.

**TABLE 3:** Predicted clustered regularly interspaced short palindromic repeats in the bacterium Gsub genome

CRISPR ID^a^	Avg length of repeats (bp)	Repeat sequence	Spacer sequence	Length of spacer (bp)
CRISPR 1	34	TGTATTGGTTTGAATATCATCAACTGTTGCTGTT	GCTGATGCTCGTAAATTATCAATTGCATCGGCATTGGTCGTTATATTGCT	50
		TGTATTGGTTTGAATATCATCAACTGTTGCTGTT	ACTGAGGTTTGTAAGGTGTTAATTGCTGTTGTATTGTTTGTAACATTACC	50
		TGTATTGGTTTGAATATCATCAACTGTTGCTGTT		
CRISPR 2	36	GTTGTGATTTGCGTTTAGGCAATAGTCTGTTACAAT	TTTGGCTATTCCTGGTTTTCTGCTCATTAG	30
		GTTGTGATTTGCGTTTAGGCAATAGTCTGTTACAAT	AAAATATATATTCAATAAAAGACAACAAAG	30
		GTTGTGATTTGCGTTTAGGCAATAGTCTGTTACAAT	CAGCAAGTTATTCTGCTAAATACATCACTG	30
		GTTGTGATTTGCGTTTAGGCAATAGTCTGTTACAAT	TTAGTATCATTTTTTACCCCCTTGTTTAAA	30
		GTTGTGATTTGCGTTTAGGCAATAGTCTGTTACAAT		
CRISPR 3	36	ATTGTAACAGACTATTGCCTAAACGCAAATCACAAC	AATGGTAGCAATGCTATTAGTTTTACAGAT	30
		ATTGTAACAGACTATTGCCTAATCGCAAATCACAAC	AAACTCACAAGGGTTAGTGATTATCGTCTT	30
		ATTGTAACAGACTATTGCCTAAACGCAAATCACAAC	TTAACTCCTTAAAGGTGCGAATATGTATAG	30
		ATTGTAACAGACTATTGCCTAAACGCAAATCACAAC	GCCTCAATTAAGCACACGGCAGGCCTACTC	30
		ATTGTAACAGACTATTGCCTAAACGCAAATCACAAC	ACAAAATTAGGCAAAGTATGAAAGAAAAA	29
		ATTGTAACAGACTATTGCCTAAACGCAAATCACAAC	CACCTTACGAATTGCTTGTTGCCAAACAA	29
		ATTGTAACAGACTATTGCCTAAACGCAAATCACAAC	TGTACAGTGCTTTTTCTGTTTCACGAATGA	30
		ATTGTAACAGACTATTGCCTAAACGCAAATCACAAC	CGAATGATTTTCATTTTGTTGTCTCCTTTA	30
		ATTGTAACAGACTATTGCCTAAACGCAAATCACAAC	CGAATGATTTTCATTTTGTTGTCTCCTTTA	30
		ATTGTAACAGACTATTGCCTAAACGCAAATCACAAC	TAATATACTTAGCTGAATAGCTCGCTGTTA	30
		ATTGTAACAGACTATTGCCTAAACGCAAATCACAAC		

a Clustered regularly interspaced palindromic repeats (CRISPR) identification (ID).

Comparison between Gsub and the two endosymbiotic bacteria Rmag and Voku revealed that Gsub contains a relatively complete set of genes encoding the type II secretion system (T2SS) (Table S4), which may mediate cellular interactions with the host. Gsub had 11 components of T2SS, including the T2SS proteins FGIJKLMN, whereas the endosymbionts Rmag and Voku do not have any of these genes. In addition, we found several toxins in Gsub, such as aureolysin, which is a proteinase that degrades bactericidal peptides from the host (54), and GTP-binding protein LepA, which is a secreted effector undermining eukaryotic trafficking and signaling pathways (55). The T2SS system is a syringe-like structure through which bacteria can secrete proteins such as enzymes into host cells (56). Although the molecules that are introduced into sponge cells by the Gsub bacterium remain unknown, the presence of T2SS suggests the potential for direct interaction between Gsub and the host sponge. This notion is consistent with the recent finding that beneficial symbionts of deep-sea hydrothermal vent mussels harbor numbers of toxin-related genes (57). However, there is evidence showing that T2SS plays a role in virulence in humans and animals, as well as in the survival of bacteria outside the host (58), consistent with the presence of T2SS in the free-living SOB.

10.1128/mSystems.00184-16.10 TABLE S4  Comparison of the genes encoding the components of type II secretion system (T2SS) in the Gsub bacterium and the reference genomes. Download TABLE S4, PDF file, 0.1 MB. Copyright © 2017 Tian et al. 2017 Tian et al. This content is distributed under the terms of the Creative Commons Attribution 4.0 International license.

### Other microbes in the sponge body.

As mentioned above, microbes in the *Thiohalorhabdales*, *Nitrosopumilus*, and *Chromatiales* also inhabit the sponge body, and they may play roles in carbon and sulfur cycling. During the genome binning process, we failed to extract the genomes of *Thiohalorhabdales* and *Chromatiales*, because they were not well separated from contigs belonging to other organisms. However, we were able to extract the genome of *Nitrosopumilus*, which is an AOA referred to below as Nsub. The Nsub genome contained 26 contigs (total length of 1,383,621 bp; total sequencing coverage of 84×) with a maximum length of 200 kbp and a mean length of 53 kbp. The genome completeness and potential contamination of the Nsub genome were estimated to be nearly 100 and 0%, respectively, based on the identification of 145 *Archaea*-specific single-copy marker genes using CheckM. Phylogenetic analysis using the 16S rRNA gene suggested that Nsub is closely related to other microbes from brine environments in the Red Sea (Fig. S6A). Nsub contains *pmoABC* genes and genes encoding copper hydroxylamine oxidoreductase and putative nitroxyl oxidoreductase, which may be responsible for the oxidation of ammonia in *Nitrosopumilus* (39). Moreover, the KEGG pathway annotation showed that Nsub is capable of carbon fixation through the hydroxypropionate-hydroxybutyrate cycle (Fig. S6B). The oxidation of ammonia could also serve to scavenge toxic ammonia from the host body. The nitrite generated may be oxidized by potential nitrite oxidizers and transformed to nitrate, which could be assimilated by the host and by other bacteria.

10.1128/mSystems.00184-16.6 FIG S6  Phylogenetic analysis and metabolic pathway for the Nsub bacterium. (A) ML unrooted tree based on the 16S rRNA genes showing the phylogenetic positions of Nsub and its close relatives. GenBank accession numbers of the reference sequences are shown. The 16S rRNA gene of Nsub displays the highest identity of 99% to an uncultured marine archaeal group 1 crenarchaeote from the brine-seawater interface of the Shaban Deep of the Red Sea. Bootstrap values of >70 are shown. (B) KEGG pathway annotation showed that the Nsub bacterium contained a nearly complete set of genes involved in carbon fixation in the hydroxypropionate-hydroxybutyrate cycle. Download FIG S6, PDF file, 0.2 MB. Copyright © 2017 Tian et al. 2017 Tian et al. This content is distributed under the terms of the Creative Commons Attribution 4.0 International license.

### Conclusions.

Here we report a potential symbiotic SOB that we term “Gsub,” living in the cold seep sponge *Suberites* sp. The bacterium is phylogenetically and functionally close to previously known intracellular symbiont SOB of vent clams. The bacterium contains complete pathways for carbon fixation and sulfide oxidation, suggesting a chemoautotrophic lifestyle. Compared to free-living SOB, Gsub and other symbiotic SOB show significant genome reduction, represented by the loss of genes for carbohydrate metabolism and respiration, cell signaling and regulation, motility and chemotaxis, stress response, and membrane transport. The loss of these genes reflects the economical use of genetic material. However, Gsub has retained special functions, including CRISPRs and protein secretion, which may have unique ecological significance in adaptation to the microenvironments of the sponge body, as these functions could not be detected in the clam symbionts. Analyses of the genome of an AOA suggest that microbes play different roles in biochemical cycles in the sponge body. These findings further our understanding of the ecological function of sponge-associated symbionts in the cold seep environment.

## MATERIALS AND METHODS

### Sponge collection.

The sponge sample was collected from Thuwal Seep II (22°16.9′N, 38°53.9′E) in the Red Sea using the remotely operated vehicle (ROV) Max Rover (Deep Sea Systems International [DSSI], USA) during cruise leg 4 organized by King Abdullah University of Science and Technology (KAUST) in May 2013. It took 3 h to recover the sample from the cold seep to the surface. Loosely attached surface bacteria were removed using 0.2-µm filtered seawater. The intact colonies were transported to the laboratory in dry ice and stored in a DNA extraction buffer (500 mM NaCl, 50 Mm Tris-HCl [pH 8], 40 mM EDTA, and 0.75 M sucrose) at −80°C until further processing.

### Two strategies of preparing materials prior to DNA extraction and sequencing.

We followed two different approaches to prepare samples before DNA extraction and sequencing.

In the first approach, the sponge tissue samples were dissected into five layers (A to E). The external layer E and internal layer A were homogenized with sterilized glass pestles in 1.5-ml centrifuge tubes for DNA extraction and metagenomic sequencing. Then, 0.8 ml of DNA extraction buffer and 10 µl of lysozyme (100 mg/ml) were added, followed by incubation at 37°C for 30 min. After that, 80 µl of SDS (20%) and 8 µl of proteinase K (10 mg/ml) were used to disrupt the cell membrane and digest proteins at 65°C for 2 h. An equal volume of phenol-chloroform-isoamyl alcohol (25:24:1) was used to denature protein, and the mixture was shaken vigorously by hand for 10 s before centrifugation at 12,000 × *g* at 4°C for 10 min. This step was repeated once using chloroform-isoamyl alcohol (24:1), and then 0.6 volume of isopropanol was added to the supernatant to precipitate DNA at −20°C for 30 min. After centrifugation at 12,000 × *g* at 4°C for 10 min, the DNA was precipitated and washed using 75% ethanol. The DNA was then dried and dissolved in double-distilled water. The DNA samples from layers A and E were subjected to library construction and sequenced using the Illumina HiSeq2000 platform in Novegene (Beijing, China). The insert size was 500 bp, while the average read length was 101 bp.

In the second approach, sponge cells and prokaryotic cells were enriched separately to generate two samples with different eukaryotic cell/prokaryotic cell ratios. In detail, a piece of intact sponge tissue (1 g [wet weight]) was homogenized in calcium- and magnesium-free seawater (CMFSW) buffer using sterilized glass pestles and 1.5-ml centrifuge tubes. The homogenate was vibrated with a vortex at the highest speed available for 5 min. The mixture of sponge cells and prokaryotic cells was collected and filtered using a polycarbonate membrane (pore size, 5.0 µm). The flowthrough (prokaryotic cell enriched) was collected, and cells were centrifuged at 10,000 × *g* for 5 min. The cells were then washed with 1 ml CMFSW before DNA extraction. The membrane (enriched in sponge cells) was washed by filtering with 10 ml of CMFSW and was then collected for DNA extraction. The sponge cell- and prokaryotic cell-enriched DNA samples were subjected to library construction and sequenced using the MiSeq platform with a library size of 500 bp and an average read length of 251 bp.

### Quality control, metagenome assembly, and genome binning.

Quality control of the HiSeq and MiSeq data sets was conducted using the NGS QC Toolkit (version 2.3) (59). Low-quality bases (Phred score of <20) at the 3′ end of each read were trimmed. Reads with an average quality score of <20 were further filtered out.

After quality control, assembly of the merged data for the layer A and E samples was performed using SPAdes (version 3.1.0) (60). The layer A/layer E metagenomes were assembled using serial kmers with sizes from 21 to 81 (step size of 10), and the results with the best contig lengths (yielded by a kmer with 71 bases) were selected for further analysis. The assembled contigs were then set as trusted contigs using the parameter “--trusted-contigs” for the reassembly of the sponge cell-enriched/prokaryote cell-enriched data sets.

Binning of prokaryotic genomes followed the differential coverage binning pipeline (61, 62) using the two metagenome pairs, and thus, two binning processes were conducted separately. In each process, the short reads of the two metagenomes after quality control were mapped to the contigs using Bowtie 2 (version 2.2.3) (63), and the sequencing coverages of the contigs in the two metagenomes were calculated using SAMtools (version 0.1.19) (64). The sequencing coverage pattern of the contigs in the two metagenomes and the tetranucleotide frequency (TNF) were used to group the contigs. Genome completeness and potential contamination were calculated using CheckM software (32).

### Community structure analysis.

The 16S rRNA genes from the assembled contigs were identified using RNAmmer (65), and the taxonomy was assigned using QIIME (version 1.7.0) (66) with the Greengenes database. The sequencing coverages of the contigs were taken into consideration in the analysis of the composition of the prokaryotic community.

### FISH experiments.

To explore the spatial location of Gsub in the sponge body, we performed fluorescence in situ hybridization (FISH) using a specific 16S rRNA-targeting probe to label the bacterium. A 20-bp probe (5′ AGGTTTAGCGGTATTGTCGC 3′) was designed with the full-length 16S rRNA gene of the Gsub. The samples used for FISH were dehydrated by sequential washing in 70%, 80%, 95%, and 100% ethanol followed by xylene (AnalaR Normapur; VWR International). The dehydrated samples were embedded in paraffin, and 5-µm sections were cut using a microtome (Leica, Germany) and placed on 0.01% poly-l-lysine-coated coverslips. Twenty microliters of distilled water was added to stretch the section, and the coverslip was air dried at 35°C for 3 h. Xylene was used to deparaffinize the sections, which were then rehydrated by serial ethanol washing. The Cy3-labeled Gsub-specific probe (20 µl; 5 ng/µl) was hybridized to the sections for 90 min at 46°C in hybridization buffer (formamide concentration of 40%). The negative control consisted of parallel sections that were stained with an unrelated probe (5′ ATTGGTCCAAGAAGTCGCC 3′) under the same conditions. A eubacterial control was set up using parallel sections stained with universal probe EUB338 (5′ GCTGCCTCCCGTAGGAGT 3′) with a formamide concentration of 30% in the hybridization buffer. All sections were washed in washing buffer for 15 min at 48°C and then were stained with 4′,6′-diamidino-2-phenylindole (DAPI) (5 ng/µl), followed by washing with 80% ethanol. The sections were then mounted using a 4:1 mixture of Citifluor and Vecta Shield immersion oil and observed under a fluorescence microscope (Olympus BX51; Olympus America).

### Phylogenetic analysis.

The ARB package (version 6.0.2) was used to locate phylogenetic positions. The SILVA database (SSURef_NR, release 119) containing 534,968 aligned 16S/18S rRNA gene sequences was used as the positional tree server. The full-length 16S rRNA gene sequence of the target Gsub was imported and aligned using the Fast aligner with the relative number of 10. The target Gsub was then added to the existing background tree to determine the phylogenetic position. The sequences of the closest relatives and representative neighboring lineages were exported as references. The sequences of the references and some BLASTn hits from the NCBI NT database, together with the target Gsub were imported into MEGA (version 6.06) (67). Gblocks analysis was used to eliminate less-informative sites in the alignments. The construction of maximum likelihood trees of 16S rRNA genes was conducted using MEGA version 6.0 with the Tamura-Nei model, the nearest-neighbor-interchange (NNI) method with 1,000 bootstrap replicates. The final alignment length was 1,303 bp. Similar procedures were used for phylogenetic analysis of the 16S rRNA gene of Nsub, and the final alignment was 1,502 bp.

The reference genes for the SoxB tree were selected by BLASTp against the online NCBI NR database. The protein sequences of the SoxB genes were aligned in MEGA version 6.0 using the Muscle algorithm with the following parameters: gap open penalty of −2.9, gap extension penalty of 0, a hydrophobicity multiplier of 1.2, UPGMB clustering method, and minimum diagonal length of 24. Alignments of the functional gene trees were subjected to Gblocks analysis to remove less-informative sites. Maximum likelihood trees were built using the Jones-Taylor-Thornton (JTT) model, NNI method with 1,000 bootstrap replicates. The model for tree building was selected based on the test of ProtTest. The final alignment length was 701 amino acids. 

A phylogenetic tree based on protein sequences of the 31 conserved single-copy genes (*tsf*, *smpB*, *rpsS*, *rpsM*, *rpsK*, *rpsJ*, *rpsI*, *rpsE*, *rpsC*, *rpsB*, *rpoB*, *rpmA*, *rplT*, *rplS*, *rplP*, *rplN*, *rplM*, *rplL*, *rplK*, *rplF*, *rplE*, *rplD*, *rplC*, *rplB*, *rplA*, *pyrG*, *pgk*, *nusA*, *infC*, *frr*, and *dnaG*) was constructed for Gsub. AMPHORA (68) was used to identify the 31 conserved single-copy genes from the genomes of Gsub and the six reference genomes. The aligned protein sequences were concatenated and imported into MEGA to construct the maximum likelihood (ML) phylogenetic tree with 1,000 bootstrap replicates. The final alignment length was 7,993 amino acids.

### Functional annotation and genomic comparison.

The tRNAs and transfer-messenger RNAs (tmRNAs) were predicted using ARAGORN (version 1.2) (69), while the rRNAs were identified using Barrnap (http://www.vicbioinformatics.com/software.barrnap.shtml). CRISPRs were identified using CRT (version 1.1) (70). Protein-coding sequences were identified using Prodigal in single-genome mode, and the coding sequences with overlapping RNA were excluded. The translated protein sequences were then annotated with BLASTp against the KEGG protein database (71), and with hmmsearch against the HMM model of Pfam (72).

The reference genomes of interest were annotated using the same protocol and then compared with Gsub. The protein sequences identified for all of the genomes were submitted to BLASTp against the nonredundant (NR) database, and the outputs were imported into MEGAN (73) for comparison with the SEED subsystem hierarchy.

### Availability of data.

All of the metagenome data have been submitted in the Sequence Read Archive (SRA) database with the accession number SRP054996. The draft genome sequences of the Gsub and Nsub bacteria have been deposited at DDBJ/EMBL/GenBank under accession numbers JYIN00000000 and LQMW00000000, respectively; the annotated genome can also be accessed at https://figshare.com/articles/New_draft_item/4236056.
