# Supplementary material for: Genome Reduction and Microbe-Host Interactions Drive Adaptation of a Sulfur-Oxidizing Bacterium Associated with a Cold Seep Sponge
Source: mSystems. 2017 Mar 21;2(2):e00184-16. doi: 10.1128/mSystems.00184-16 (PMC5361782; doi:10.1128/mSystems.00184-16)
Supplement: TABLE S1 [file sys002172098st7.pdf]

Table S1

	HiSeq-sponge				Miseq-sponge				Hiseq-water	
	Layer A read 1	Layer A read 2	layer E read 1	layer E read 2	Sponge cell-enriched read 1	Sponge cell-enriched read 2	Prokaryote cell-enriched read 1	Prokaryote cell-enriched read 2	Water-read 1	Water-read 2
Average read length	101	101	101	101	251	251	251	251	101	101
Total number of reads	21,862,940	21,862,940	22,273,888	22,273,888	8,397,949	8,397,949	8,572,844	8,572,844	14,312,330	14,312,330
Total number of reads with non-ATGC bases	2,890	12,488	2,848	12,583	3,843	1,337	3,246	1,201	20,105	9,409
Percentage of reads with non-ATGC bases	0.0%	0.1%	0.0%	0.1%	0.05%	0.02%	0.04%	0.01%	0.14%	0.07%
Total number of bases	1,989,527,540	1,989,527,540	2,026,923,808	2,026,923,808	2,436,127,659	2,440,072,840	2,444,814,526	2,448,375,663	1,445,545,330	1,445,545,330
Total number of high quality bases	1,965,114,822	1,953,547,897	2,002,583,624	1,989,862,693	2,400,920,059	2,141,171,615	2,409,342,382	2,129,364,878	1,437,434,961	1,388,052,782
Percentage of high quality bases	98.8%	98.2%	98.8%	98.2%	98.55%	87.75%	98.55%	86.97%	99.44%	96.02%
Total contigs after assembly	41,045								75,428	
Total bases after assembly	131,911,312								88,496,419	
N50 length of contigs (bp)	7,480								3,144	
