# Supplementary figures and images for: Genome Reduction and Microbe-Host Interactions Drive Adaptation of a Sulfur-Oxidizing Bacterium Associated with a Cold Seep Sponge
Source: mSystems. 2017 Mar 21;2(2):e00184-16. doi: 10.1128/mSystems.00184-16 (PMC5361782; doi:10.1128/mSystems.00184-16)

**Figure S1**

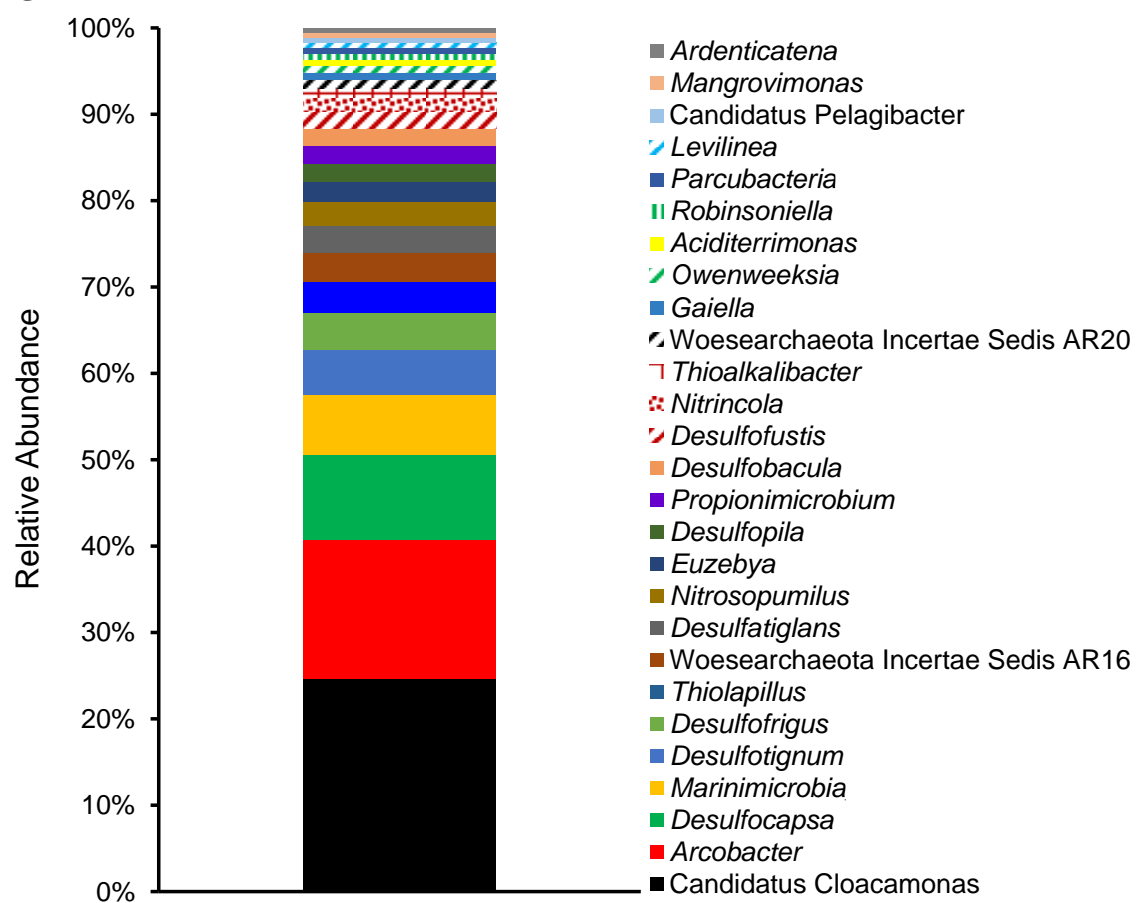

Supplement: FIG S1 [file sys002172098sf1.pdf]

**Figure S2**

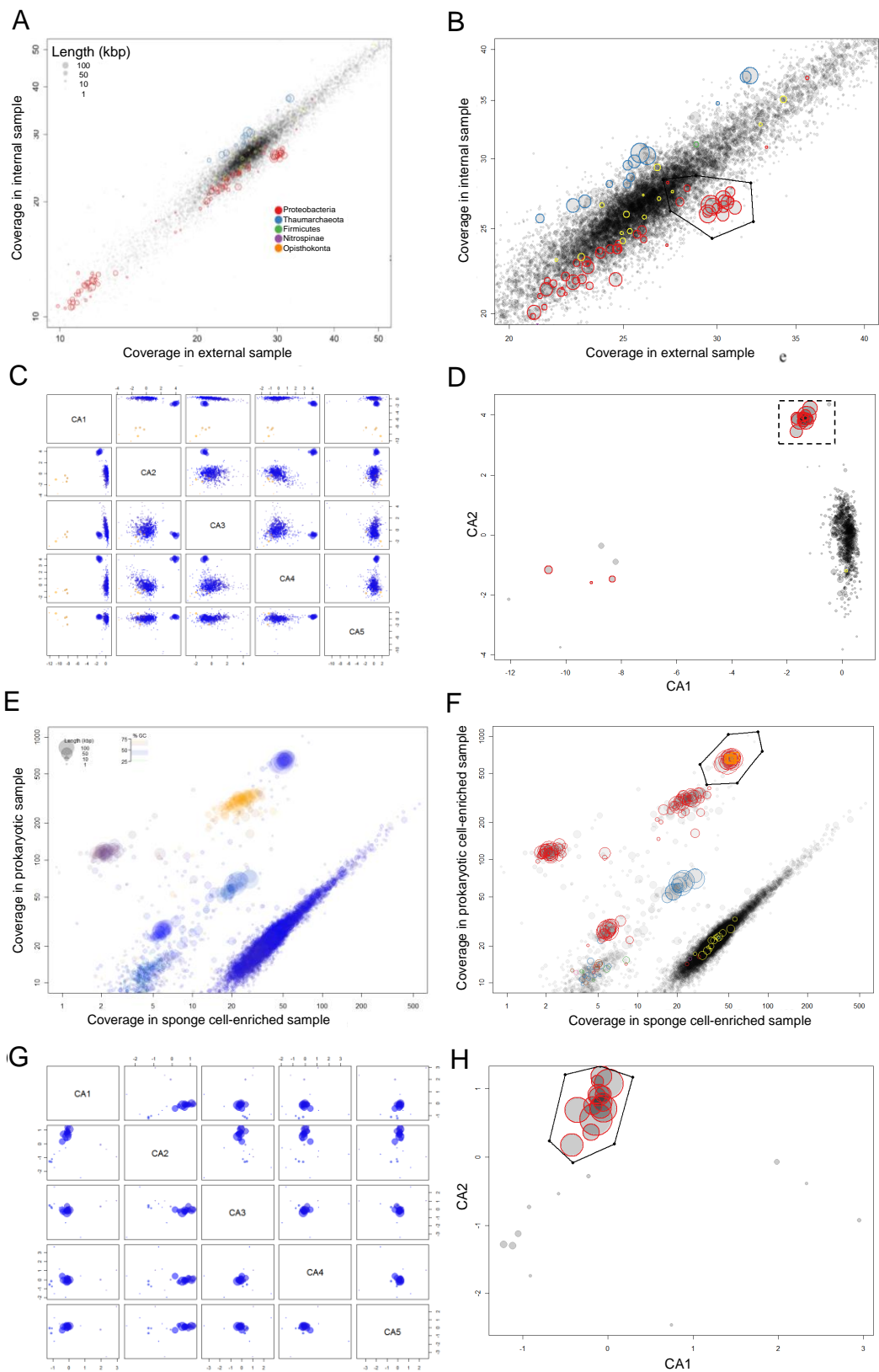

Supplement: FIG S2 [file sys002172098sf2.pdf]

**Figure S3**

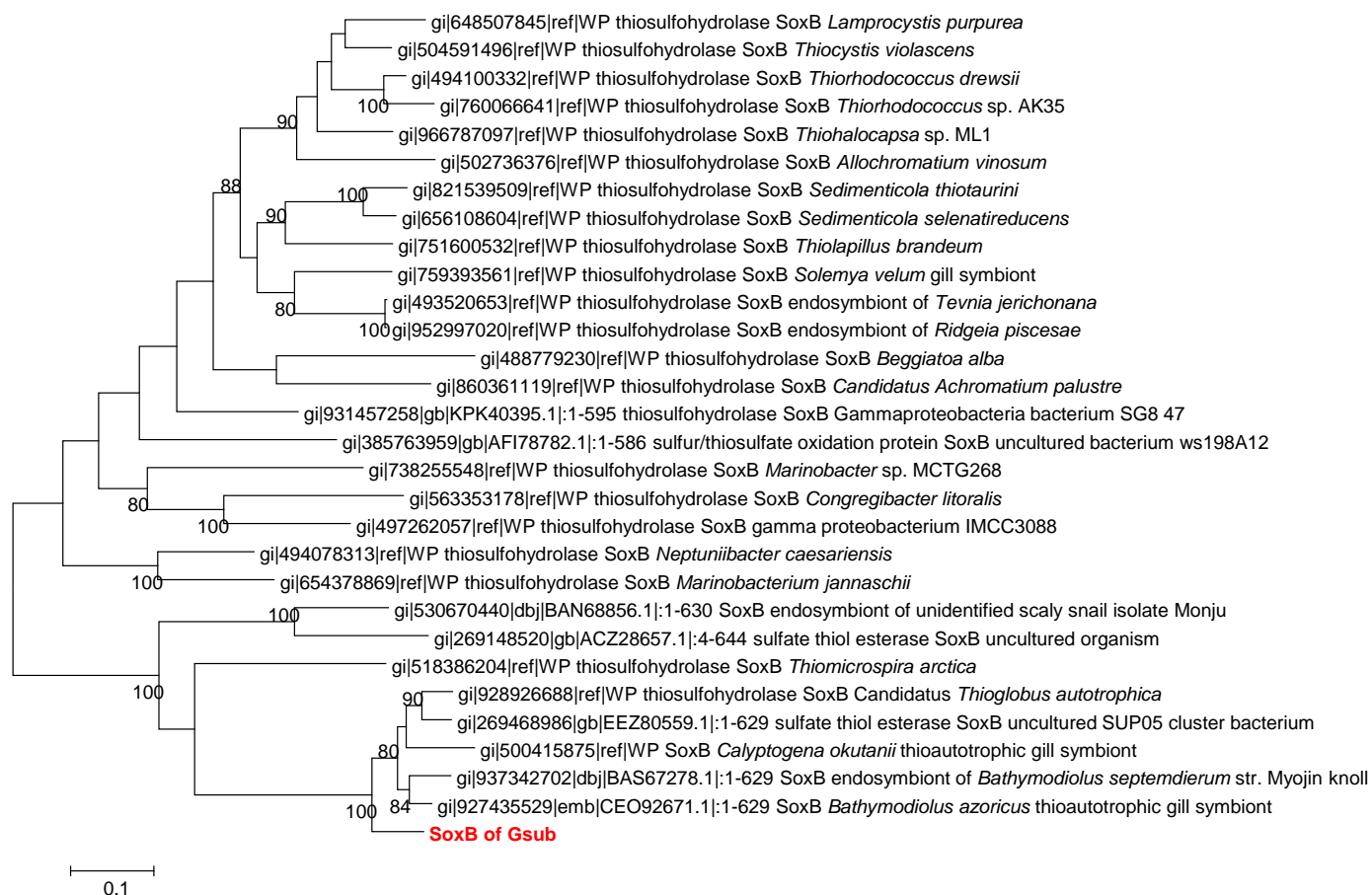

Supplement: FIG S3 [file sys002172098sf3.pdf]

Figure S4

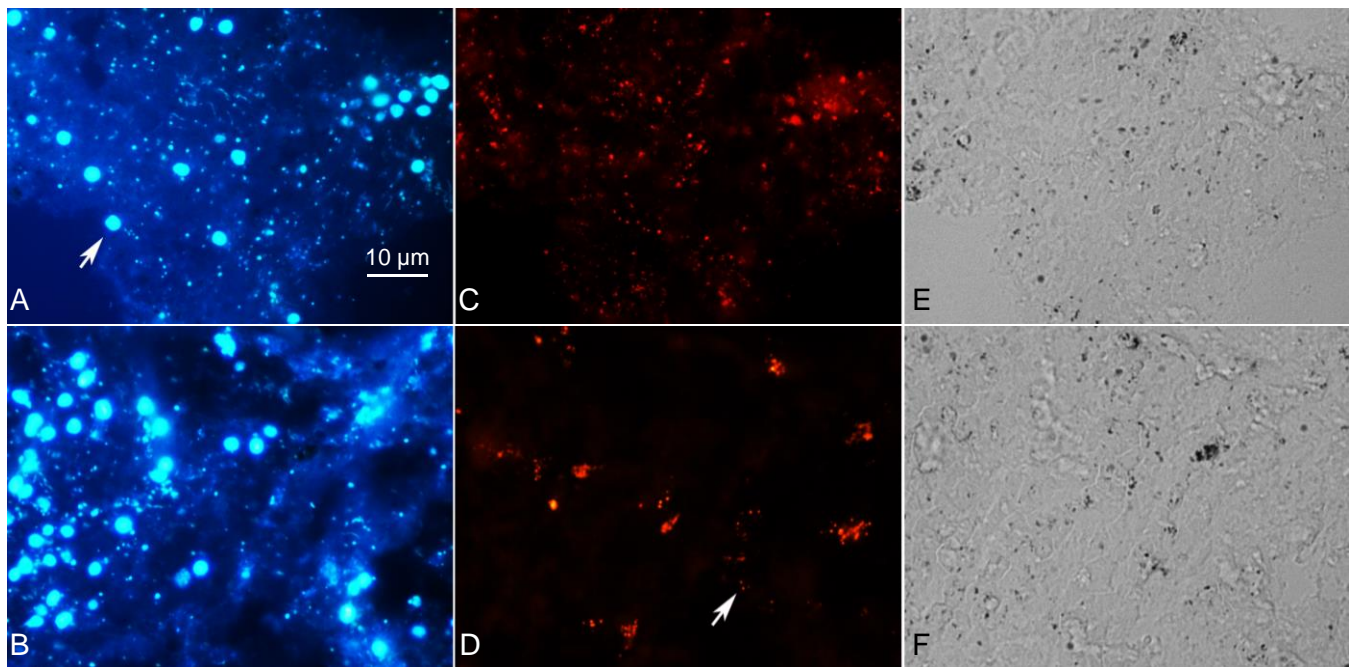

Supplement: FIG S4 [file sys002172098sf4.pdf]

Figure S5

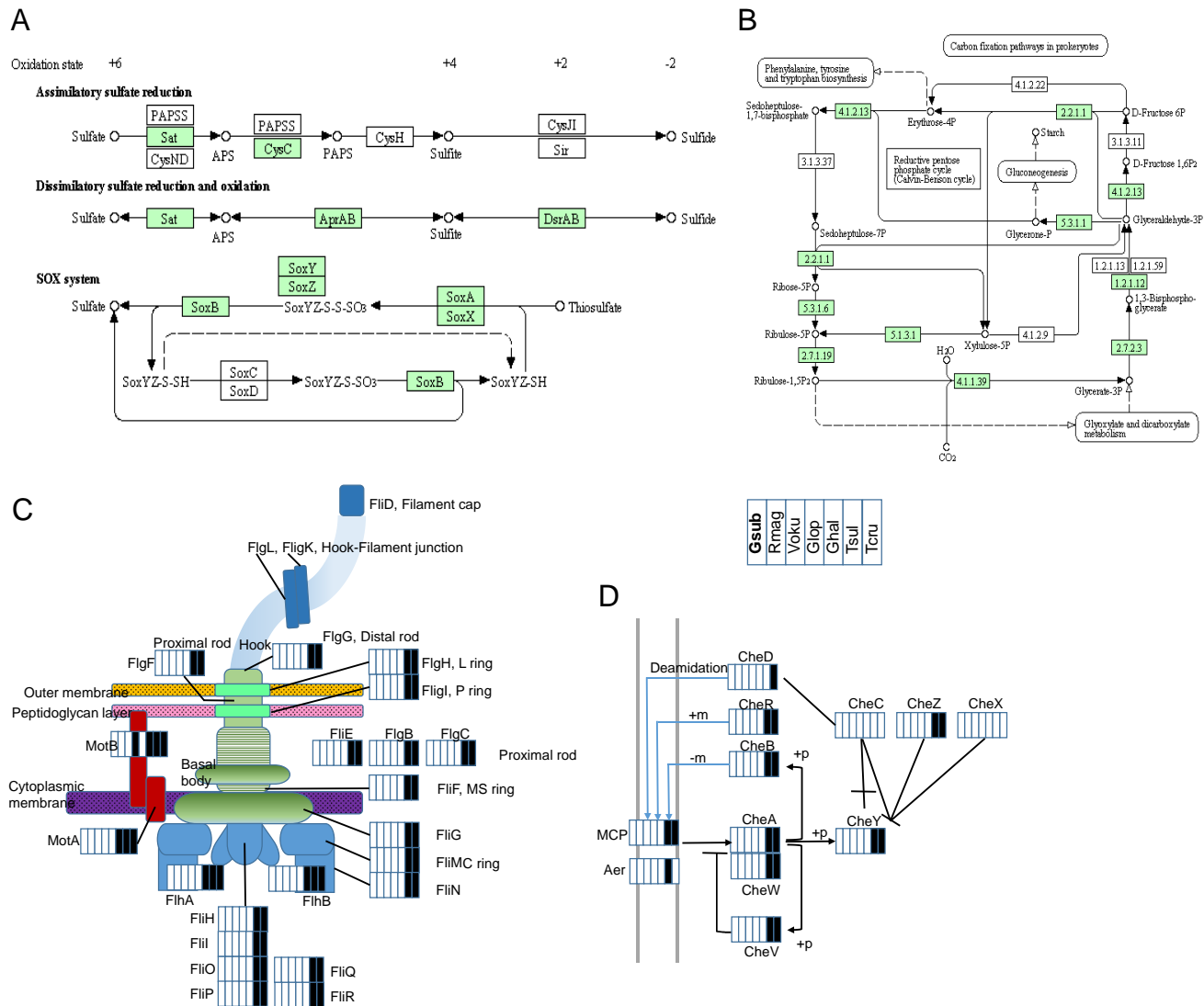

Supplement: FIG S5 [file sys002172098sf5.pdf]
