# Supplementary material for: Genome Reduction and Microbe-Host Interactions Drive Adaptation of a Sulfur-Oxidizing Bacterium Associated with a Cold Seep Sponge
Source: mSystems. 2017 Mar 21;2(2):e00184-16. doi: 10.1128/mSystems.00184-16 (PMC5361782; doi:10.1128/mSystems.00184-16)
Supplement: TABLE S2 [file sys002172098st8.pdf]

Table S2

Pfam	Annotation	Gsub	Rma g	Voku	Glop	Ghal	Tsul	Tcru
PF03485	Arg_tRNA_synt_N Arginyl tRNA synthetase N terminal domain	1	1	1	1	1	1	1
PF03484	B5 tRNA synthetase B5 domain	1	1	1	1	1	1	1
PF01121	P-loop_NTPase CoaE Dephospho-CoA kinase	1	1	1	1	1	1	1
PF03772	Competence Competence protein	1	0	0	1	1	1	1
PF03602	NADP_Rossmann Cons_hypoth95 Conserved hypothetical protein 95	1	1	1	1	1	1	1
PF06418	P-loop_NTPase CTP_synth_N CTP synthase N-terminus	1	1	1	1	1	1	1
PF02224	P-loop_NTPase Cytidylate_kin Cytidylate kinase	1	1	1	1	1	1	1
PF00712	DNA_clamp DNA_pol3_beta DNA polymerase III beta subunit, N-terminal domain	1	1	1	1	1	1	1
PF02767	DNA_clamp DNA_pol3_beta_2 DNA polymerase III beta subunit, central domain	1	1	1	1	1	1	1
PF02768	DNA_clamp DNA_pol3_beta_3 DNA polymerase III beta subunit, C-terminal domain	1	1	1	1	1	1	1
PF00035	DSRM dsrm Double-stranded RNA binding motif	1	1	1	1	1	1	1
PF00889	EF_TS Elongation factor TS	1	1	1	1	1	1	1
PF01176	OB eIF-1a Translation initiation factor 1A / IF-1	1	1	1	1	1	2	1
PF00113	Enolase_TIM Enolase_C Enolase, C-terminal TIM barrel domain	1	1	1	1	1	1	1
PF03952	Enolase_N Enolase_N Enolase, N-terminal domain	1	1	1	1	1	1	1
PF06574	HUP FAD_syn FAD synthetase	1	1	1	1	1	1	1

PF03147	FDX-ACB Ferredoxin-fold anticodon binding domain	1	1	1	1	1	1	1
PF01687	Flavokinase Riboflavin kinase	1	1	1	1	1	1	1
PF02938	GAD GAD GAD domain	1	1	1	1	1	1	1
PF02527	NADP_Rossmann GidB rRNA small subunit methyltransferase G	1	1	0	1	1	1	1
PF00958	GMP_synt_C GMP synthase C terminal domain	1	1	1	1	1	1	1
PF01025	GrpE GrpE	1	1	1	1	1	1	1
PF01018	GTP1_OBG GTP1/OBG	1	1	1	1	1	1	1
PF11987	IF-2 Translation-initiation factor 2	1	1	1	1	1	1	1
PF04760	HTH IF2_N Translation initiation factor IF-2, N-terminal region	1	1	1	1	1	1	1
PF00707	IF3_C Translation initiation factor IF-3, C-terminal domain	1	1	1	1	1	1	1
PF05198	IF3_N Translation initiation factor IF-3, N-terminal domain	1	1	1	1	1	1	0
PF01715	P-loop_NTPase IPPT IPP transferase	1	1	1	1	1	1	1
PF06421	LepA_C GTP-binding protein LepA C-terminus	1	1	1	1	1	1	1
PF01795	NADP_Rossmann Methyltransf_5 MraW methylase family	1	1	1	1	1	1	1
PF02873	MurB_C UDP-N-acetylenolpyruvoylglucosamine reductase, C-terminal domain	1	1	1	1	1	1	1
PF08529	NusA_N NusA N-terminal domain	1	1	1	1	1	1	1
PF02410	NTP_transf RsfS Ribosomal silencing factor during starvation	1	1	0	1	1	1	1
PF01195	Pept_tRNA_hydro Peptidyl-tRNA hydrolase	1	1	1	1	1	1	1
PF01252	Peptidase_A8 Signal peptidase (SPase) II	1	1	1	1	1	2	1
PF00162	PGK Phosphoglycerate kinase	1	1	1	1	1	1	1

PF02912	tRNA_bind_arm Phe_tRNA-synt_N Aminoacyl tRNA synthetase class II, N-terminal domain	1	1	1	1	1	1	1
PF03726	PNPase Polyribonucleotide nucleotidyltransferase, RNA binding domain	1	1	1	1	1	1	1
PF01416	PseudoU_synth_1 tRNA pseudouridine synthase	1	1	1	0	1	1	1
PF02033	RBFA Ribosome-binding factor A	1	1	1	1	1	1	1
PF00154	P-loop_NTPase RecA recA bacterial DNA recombination protein	1	0	0	1	2	1	1
PF02132	RecR RecR protein	1	0	0	1	1	1	1
PF00825	S5 Ribonuclease_P Ribonuclease P	1	1	1	1	1	1	1
PF00687	Ribosomal_L1 Ribosomal protein L1p/L10e family	1	1	1	1	1	1	1
PF00466	Ribosomal_L10 Ribosomal protein L10	1	1	1	1	1	1	1
PF00298	Ribosomal_L11 Ribosomal protein L11, RNA binding domain	1	1	1	1	1	1	1
PF03946	Ribosomal_L11_N Ribosomal protein L11, N-terminal domain	1	1	1	1	1	1	1
PF00542	Ribosomal_L12 Ribosomal protein L7/L12 C-terminal domain	1	1	1	1	1	1	1
PF00572	Ribosomal_L13 Ribosomal protein L13	1	1	1	1	1	1	1
PF00238	Ribosomal_L14 Ribosomal protein L14p/L23e	1	1	1	1	1	1	1
PF00252	Ribosomal_L16 Ribosomal protein L16p/L10e	1	1	1	1	1	1	1
PF01196	Ribosomal_L17 Ribosomal protein L17	1	1	1	1	1	1	1
PF00828	Ribos_L15p_L18e Ribosomal_L27A Ribosomal proteins 50S-L15, 50S-L18e, 60S-L27A	1	1	1	1	1	1	1
PF00861	S11_L18p Ribosomal_L18p Ribosomal L18 of archaea, bacteria,	1	1	1	1	1	1	1

	mitoch. and chloroplast							
PF01245	KOW Ribosomal_L19 Ribosomal protein L19	1	1	1	1	1	1	1
PF00181	OB Ribosomal_L2 Ribosomal Proteins L2, RNA binding domain	1	1	1	1	1	1	1
PF03947	KOW Ribosomal_L2_C Ribosomal Proteins L2, C-terminal domain	1	1	1	1	1	1	1
PF00453	Ribosomal_L20 Ribosomal protein L20	1	1	1	1	1	1	1
PF00829	Ribosomal_L21p Ribosomal prokaryotic L21 protein	1	1	1	1	1	1	1
PF00237	Ribosomal_L22 Ribosomal protein L22p/L17e	1	1	1	1	1	1	1
PF00276	Ribosomal_L23 Ribosomal protein L23	1	1	1	1	1	1	1
PF01016	Ribosomal_L27 Ribosomal L27 protein	1	1	1	1	1	1	1
PF00830	Ribosomal_L28 Ribosomal L28 family	1	1	1	1	1	1	1
PF00831	Ribo_L29 Ribosomal_L29 Ribosomal L29 protein	1	1	1	1	1	1	1
PF00297	EFTPs Ribosomal_L3 Ribosomal protein L3	1	1	1	1	1	1	1
PF01783	Zn_Beta_Ribbon Ribosomal_L32p Ribosomal L32p protein family	1	1	1	0	1	1	1
PF01632	Ribosomal_L35p Ribosomal protein L35	1	1	1	1	0	1	1
PF00573	Ribosomal_L4 Ribosomal protein L4/L1 family	1	1	1	1	1	1	1
PF00281	Ribosomal_L5 Ribosomal protein L5	1	1	1	1	1	1	1
PF00673	Ribosomal_L5_C ribosomal L5P family C-terminus	1	1	1	1	1	1	1
PF00347	Ribosomal_L6 Ribosomal protein L6	1	1	1	1	1	1	1
PF03948	Ribosomal_L9_C Ribosomal protein L9, C-terminal domain	1	1	1	1	1	1	1

PF01281	Ribosomal_L9_N Ribosomal protein L9, N-terminal domain	1	1	1	1	1	1	1
PF00338	Ribosomal_S10 Ribosomal protein S10p/S20e	1	1	1	1	1	1	1
PF00411	S11_L18p Ribosomal_S11 Ribosomal protein S11	1	1	1	1	1	1	1
PF00164	OB Ribosom_S12_S23 Ribosomal protein S12/S23	1	1	1	0	1	1	1
PF00416	H2TH Ribosomal_S13 Ribosomal protein S13/S18	1	1	1	1	1	1	1
PF00312	S15_NS1 Ribosomal_S15 Ribosomal protein S15	1	1	1	1	1	1	1
PF00886	Ribosomal_S16 Ribosomal protein S16	1	1	1	1	1	1	1
PF00366	OB Ribosomal_S17 Ribosomal protein S17	1	1	1	1	1	1	1
PF01084	Ribosomal_S18 Ribosomal protein S18	1	1	1	1	1	1	1
PF00203	Ribosomal_S19 Ribosomal protein S19	1	1	1	1	1	1	1
PF00318	Ribosomal_S2 Ribosomal protein S2	1	1	1	1	1	1	1
PF01649	Ribosomal_S20p Ribosomal protein S20	1	1	1	0	1	1	1
PF00189	Ribosomal_S3_C Ribosomal protein S3, C-terminal domain	1	1	1	1	1	1	1
PF00163	S4 Ribosomal_S4 Ribosomal protein S4/S9 N-terminal domain	1	1	1	1	1	1	1
PF00333	DSRM Ribosomal_S5 Ribosomal protein S5, N-terminal domain	1	1	1	1	1	1	1
PF03719	S5 Ribosomal_S5_C Ribosomal protein S5, C-terminal domain	1	1	1	1	1	1	1
PF01250	Ribosomal_S6 Ribosomal protein S6	1	1	1	1	1	1	1
PF00177	Ribosomal_S7 Ribosomal protein S7p/S5e	1	1	1	1	1	1	1
PF00410	Ribosomal_S8 Ribosomal protein S8	1	1	1	1	1	1	1

PF00380	S5 Ribosomal_S9 Ribosomal protein S9/S16	1	1	1	1	1	1	1
PF01782	EFTPs RimM RimM N-terminal domain	1	1	1	1	1	1	1
PF01000	RNA_pol_A_bac RNA polymerase Rpb3/RpoA insert domain	1	1	1	1	1	1	1
PF03118	HHH RNA_pol_A_CTD Bacterial RNA polymerase, alpha chain C terminal domain	1	1	1	1	1	1	1
PF01193	RBP11-like RNA_pol_L RNA polymerase Rpb3/Rpb11 dimerisation domain	1	1	1	1	1	1	1
PF04997	RNA_pol_Rpb1_1 RNA polymerase Rpb1, domain 1	1	1	1	1	1	1	1
PF00623	RNA_pol_Rpb1_2 RNA polymerase Rpb1, domain 2	1	1	1	1	1	1	1
PF04983	RNA_pol_Rpb1_3 RNA polymerase Rpb1, domain 3	1	1	1	1	1	1	1
PF05000	RNA_pol_Rpb1_4 RNA polymerase Rpb1, domain 4	1	1	1	1	1	1	1
PF04998	RNA_pol_Rpb1_5 RNA polymerase Rpb1, domain 5	1	1	1	1	1	1	1
PF04563	RNA_pol_Rpb2_1 RNA polymerase beta subunit	1	1	1	1	1	1	1
PF04561	RNA_pol_Rpb2_2 RNA polymerase Rpb2, domain 2	1	1	1	1	1	1	1
PF04565	RNA_pol_Rpb2_3 RNA polymerase Rpb2, domain 3	1	1	1	1	1	1	1
PF10385	RNA_pol_Rpb2_45 RNA polymerase beta subunit external 1 domain	1	1	1	1	1	1	1
PF00562	LEF-8-like RNA_pol_Rpb2_6 RNA polymerase Rpb2, domain 6	1	1	1	1	1	1	1
PF04560	RNA_pol_Rpb2_7 RNA polymerase Rpb2, domain 7	1	1	1	1	1	1	1
PF01765	RRF Ribosome recycling factor	1	1	1	1	1	1	1
PF07499	UBA RuvA_C RuvA, C-terminal	1	0	1	1	1	1	1

	domain							
PF01330	OB RuvA_N RuvA N terminal domain	1	0	1	1	1	1	1
PF05491	HTH RuvB_C Holliday junction DNA helicase ruvB C-terminus	1	0	1	1	1	1	1
PF02773	S-AdoMet_synt_C S-adenosylmethionine synthetase, C-terminal domain	1	1	1	1	1	2	1
PF02772	S-AdoMet_synt_M S-adenosylmethionine synthetase, central domain	1	1	1	1	1	2	1
PF00584	SecE SecE/Sec61-gamma subunits of protein translocation complex	1	1	1	1	1	1	1
PF03840	SecG Preprotein translocase SecG subunit	1	1	1	1	1	1	1
PF00344	SecY SecY translocase	1	1	1	1	1	1	1
PF02403	tRNA_bind_arm Seryl_tRNA_N Seryl-tRNA synthetase N-terminal domain	1	1	1	1	1	1	1
PF01668	SmpB SmpB protein	1	1	1	1	1	1	1
PF02978	SRP_SPB Signal peptide binding domain	1	1	1	1	1	1	1
PF00763	AA_dh_N THF_DHG_CYH Tetrahydrofolate dehydrogenase/cyclohydrolase, catalytic domain	1	1	1	1	1	1	2
PF02882	NADP_Rossmann THF_DHG_CYH_C Tetrahydrofolate dehydrogenase/cyclohydrolase, NAD(P)-binding domain	1	1	1	1	1	1	2
PF00121	TIM_barrel TIM Triosephosphate isomerase	1	1	1	1	1	1	1
PF08275	Toprim_N DNA primase catalytic core, N-terminal domain	1	1	1	1	1	1	1
PF03461	TRCF TRCF domain	1	1	1	1	1	1	1
PF05698	Trigger_C Trigger_C Bacterial trigger	1	1	1	1	1	1	1

	factor protein (TF) C-terminus							
PF05697	Trigger_N Bacterial trigger factor protein (TF)	1	1	1	1	1	1	1
PF01746	SPOUT tRNA_m1G_MT tRNA (Guanine-1)-methyltransferase	1	1	1	1	1	1	1
PF00750	HUP tRNA-synt_1d tRNA synthetases class I (R)	1	1	1	1	1	2	2
PF01409	tRNA_synt_II tRNA-synt_2d tRNA synthetases class II core domain (F)	1	1	1	1	1	1	1
PF01509	TruB_N TruB family pseudouridylate synthase (N terminal domain)	1	1	1	1	1	1	1
PF00627	UBA UBA UBA/TS-N domain	1	1	1	1	1	1	1
PF02130	UPF0054 Uncharacterized protein family UPF0054	1	1	1	1	1	1	1
PF02367	P-loop_NTPase TsaE Threonylcarbamoyl adenosine biosynthesis protein TsaE	1	1	1	1	1	1	1
PF03652	YqgF RuvX Holliday junction resolvase	1	1	1	1	1	1	1
PF12344	UvrB Ultra-violet resistance protein B	1	1	0	1	1	1	1
PF08459	Endonuclease UvrC_HhH_N UvrC Helix-hairpin-helix N-terminal	1	1	0	1	1	1	1
PF10458	tRNA_bind_arm Val_tRNA-synt_C Valyl tRNA synthetase tRNA binding arm	1	1	1	1	1	1	1
PF06071	Ubiquitin YchF-GTPase_C Protein of unknown function (DUF933)	1	1	1	1	1	1	1
PF06689	zf-C4_ClpX ClpX C4-type zinc finger	1	1	1	1	1	1	1
