# Supplementary material for: Genome Reduction and Microbe-Host Interactions Drive Adaptation of a Sulfur-Oxidizing Bacterium Associated with a Cold Seep Sponge
Source: mSystems. 2017 Mar 21;2(2):e00184-16. doi: 10.1128/mSystems.00184-16 (PMC5361782; doi:10.1128/mSystems.00184-16)
Supplement: TABLE S3 [file sys002172098st9.pdf]

Table S3

Unique KEGGs for Gsub when compared with Rmag, Voku, Tcru and Tsul	
ko:K14986	*fixL*; two-component system, LuxR family, sensor kinase FixL [EC:2.7.13.3]
ko:K00860	*cysC*; adenylylsulfate kinase [EC:2.7.1.25]
ko:K09952	*csn1*, *cas9*; CRISPR-associated endonuclease Csn1 [EC:3.1.-.-]
ko:K07516	*fadN*; 3-hydroxyacyl-CoA dehydrogenase [EC:1.1.1.35]
ko:K02028	ABC.PA.A; polar amino acid transport system ATP-binding protein [EC:3.6.3.21]
ko:K09969	*aapJ*, *bztA*; general L-amino acid transport system substrate-binding protein
ko:K02315	*dnaC*; DNA replication protein DnaC
ko:K00368	*nirK*; nitrite reductase (NO-forming) [EC:1.7.2.1]
ko:K00743	GGTA1; N-acetyllactosaminide 3-alpha-galactosyltransferase [EC:2.4.1.87]
ko:K00590	E2.1.1.113; site-specific DNA-methyltransferase (cytosine-N4-specific) [EC:2.1.1.113]
ko:K00721	DPM1; dolichol-phosphate mannosyltransferase [EC:2.4.1.83]
ko:K09971	*aapM*, *bztC*; general L-amino acid transport system permease protein
ko:K06350	*kipA*; antagonist of KipI
ko:K00632	*fadA*, *fadI*; acetyl-CoA acyltransferase [EC:2.3.1.16]
ko:K02305	*norC*; nitric oxide reductase subunit C
ko:K07160	K07160; UPF0271 protein
ko:K02843	*waaF*, *rfaF*; heptosyltransferase II [EC:2.4.-.-]
ko:K06445	*fadE*; acyl-CoA dehydrogenase [EC:1.3.99.-]
ko:K00571	E2.1.1.72; site-specific DNA-methyltransferase (adenine-specific) [EC:2.1.1.72]
ko:K07638	*envZ*; two-component system, OmpR family, osmolarity sensor histidine kinase EnvZ [EC:2.7.13.3]
ko:K01401	*aur*; aureolysin [EC:3.4.24.29]

ko:K02196	*ccmD*; heme exporter protein D
ko:K07493	K07493; putative transposase
ko:K13444	SUMF1, FGE; sulfatase modifying factor 1
ko:K07146	K07146; UPF0176 protein
ko:K00449	*pcaH*; protocatechuate 3,4-dioxygenase, beta subunit [EC:1.13.11.3]
ko:K04561	*norB*; nitric oxide reductase subunit B [EC:1.7.2.5]
ko:K00558	DNMT1, dcm; DNA (cytosine-5)-methyltransferase 1 [EC:2.1.1.37]
ko:K03885	*ndh*; NADH dehydrogenase [EC:1.6.99.3]
ko:K07319	*yhdJ*; adenine-specific DNA-methyltransferase [EC:2.1.1.72]
ko:K09970	*aapQ*, *bztB*; general L-amino acid transport system permease protein
ko:K00113	*glpC*; glycerol-3-phosphate dehydrogenase subunit C [EC:1.1.5.3]
ko:K01359	PCSK1; proprotein convertase subtilisin/kexin type 1 [EC:3.4.21.93]
ko:K00718	FUT1_2; galactoside 2-L-fucosyltransferase 1/2 [EC:2.4.1.69]
ko:K12264	*norV*; anaerobic nitric oxide reductase flavorubredoxin
ko:K01247	*alkA*; DNA-3-methyladenine glycosylase II [EC:3.2.2.21]
ko:K06996	K06996; uncharacterized protein
ko:K09678	HS3ST4; [heparan sulfate]-glucosamine 3-sulfotransferase 4 [EC:2.8.2.-]
ko:K12984	*waaE*, *kdtX*; (heptosyl) LPS beta-1,4-glucosyltransferase [EC:2.4.1.-]
**Unique KEGGs for Gsub when compared with Glop, Ghal, Tcru and Tsul**	
ko:K14986	*fixL*; two-component system, LuxR family, sensor kinase FixL [EC:2.7.13.3]
ko:K09952	*csn1*, *cas9*; CRISPR-associated endonuclease Csn1 [EC:3.1.-.-]
ko:K02315	*dnaC*; DNA replication protein DnaC
ko:K00721	DPM1; dolichol-phosphate mannosyltransferase [EC:2.4.1.83]
ko:K01147	*rnb*; exoribonuclease II [EC:3.1.13.1]
ko:K07493	K07493; putative transposase
ko:K00449	*pcaH*; protocatechuate 3,4-dioxygenase, beta subunit [EC:1.13.11.3]
ko:K01571	*oadA*; oxaloacetate decarboxylase, alpha subunit [EC:4.1.1.3]

ko:K13012	*wbqP*; O-antigen biosynthesis protein WbqP
ko:K09765	K09765; uncharacterized protein
ko:K04561	*norB*; nitric oxide reductase subunit B [EC:1.7.2.5]
ko:K01359	PCSK1; proprotein convertase subtilisin/kexin type 1 [EC:3.4.21.93]
ko:K12984	*waaE*, *kdtX*; (heptosyl) LPS beta-1,4-glucosyltransferase [EC:2.4.1.-]
ko:K00936	E2.7.3.-
ko:K00860	*cysC*; adenylylsulfate kinase [EC:2.7.1.25]
ko:K07400	*nfuA*; Fe/S biogenesis protein NfuA
ko:K00368	*nirK*; nitrite reductase (NO-forming) [EC:1.7.2.1]
ko:K00743	GGTA1; N-acetyllactosaminide 3-alpha-galactosyltransferase [EC:2.4.1.87]
ko:K01141	*sbcB*, *exoI*; exodeoxyribonuclease I [EC:3.1.11.1]
ko:K00632	*fadA*, *fadI*; acetyl-CoA acyltransferase [EC:2.3.1.16]
ko:K02305	*norC*; nitric oxide reductase subunit C
ko:K07638	*envZ*; two-component system, OmpR family, osmolarity sensor histidine kinase EnvZ [EC:2.7.13.3]
ko:K01802	E5.2.1.8; peptidylprolyl isomerase [EC:5.2.1.8]
ko:K01401	*aur*; aureolysin [EC:3.4.24.29]
ko:K02196	*ccmD*; heme exporter protein D
ko:K03184	*ubiF*; 2-octaprenyl-3-methyl-6-methoxy-1,4-benzoquinol hydroxylase [EC:1.14.13.-]
ko:K01572	*oadB*; oxaloacetate decarboxylase, beta subunit [EC:4.1.1.3]
ko:K01676	E4.2.1.2A, fumA, fumB; fumarate hydratase, class I [EC:4.2.1.2]
ko:K01493	*comEB*; dCMP deaminase [EC:3.5.4.12]
ko:K03885	*ndh*; NADH dehydrogenase [EC:1.6.99.3]
ko:K14998	SURF1, SHY1; surfeit locus 1 family protein
ko:K06077	*slyB*; outer membrane lipoprotein SlyB
ko:K00718	FUT1_2; galactoside 2-L-fucosyltransferase 1/2 [EC:2.4.1.69]

ko:K09678	HS3ST4; [heparan sulfate]-glucosamine 3-sulfotransferase 4 [EC:2.8.2.-]
