# Supplementary material for: Genome Reduction and Microbe-Host Interactions Drive Adaptation of a Sulfur-Oxidizing Bacterium Associated with a Cold Seep Sponge
Source: mSystems. 2017 Mar 21;2(2):e00184-16. doi: 10.1128/mSystems.00184-16 (PMC5361782; doi:10.1128/mSystems.00184-16)
Supplement: TABLE S4 [file sys002172098st10.pdf]

Table S4

Pfam	Description	**Gsub**	Rmag	Voku	Glop	Ghal	Tsul	Tcru
PF00263.16	Bacterial type II and III secretion system protein	2	0	0	0	2	3	3
PF03958.12	Bacterial type II/III secretion system short domain	3	0	0	0	1	3	3
PF00437.15	Type II/IV secretion system protein	2	0	0	0	3	4	4
PF00482.18	Type II secretion system (T2SS), protein F	2	0	0	0	0	0	0
PF08334.6	Type II secretion system (T2SS), protein G	1	0	0	0	0	1	1
PF02501.12	Type II secretion system (T2SS), protein I	1	0	0	0	0	0	0
PF11612.3	Type II secretion system (T2SS), protein J	1	0	0	0	0	1	1
PF03934.8	Type II secretion system (T2SS), protein K	1	0	0	0	0	0	0
PF05134.8	Type II secretion system (T2SS), protein L	1	0	0	0	0	1	1
PF04612.7	Type II secretion system (T2SS), protein M	1	0	0	0	0	1	1
PF01203.14	Type II secretion system (T2SS), protein N	1	0	0	0	0	1	1
