# Supplementary material for: Genome Reduction and Microbe-Host Interactions Drive Adaptation of a Sulfur-Oxidizing Bacterium Associated with a Cold Seep Sponge
Source: mSystems. 2017 Mar 21;2(2):e00184-16. doi: 10.1128/mSystems.00184-16 (PMC5361782; doi:10.1128/mSystems.00184-16)
Supplement: FIG S6 [file sys002172098sf6.pdf]

Figure S6

A

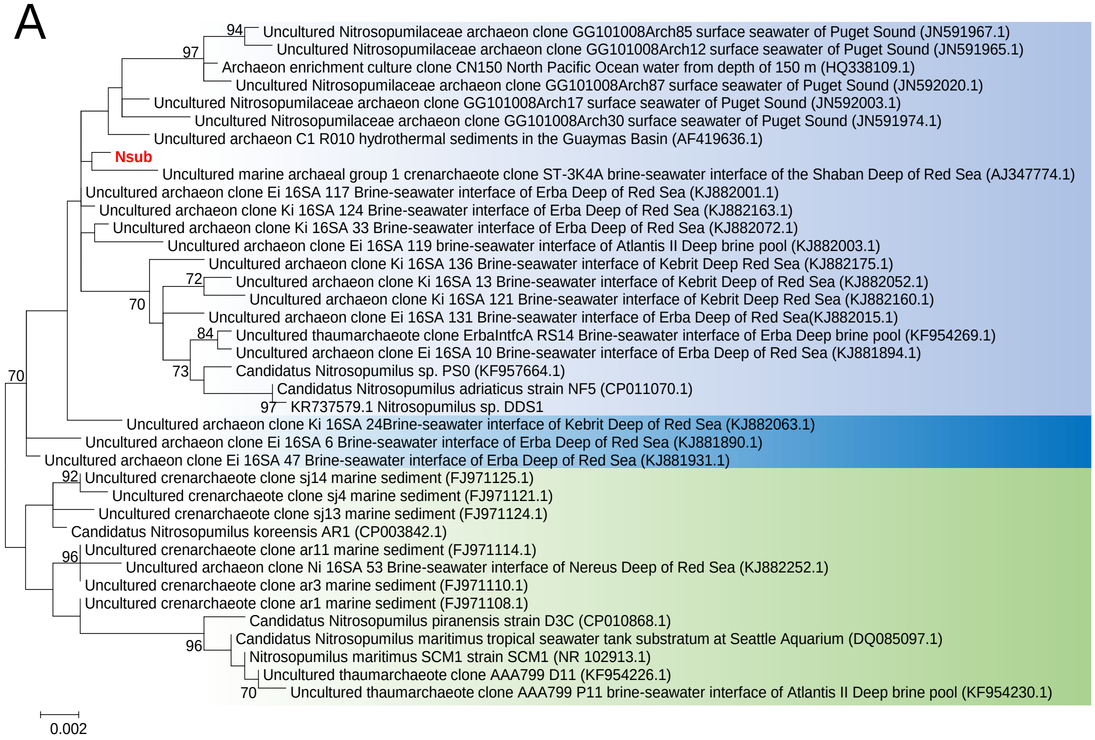

Uncultured Nitrosopumilaceae archaeon clone GG101008Arch85 surface seawater of Puget Sound (JN591967.1)
Uncultured Nitrosopumilaceae archaeon clone GG101008Arch12 surface seawater of Puget Sound (JN591965.1)
Archaeon enrichment culture clone CN150 North Pacific Ocean water from depth of 150 m (HQ338109.1)
Uncultured Nitrosopumilaceae archaeon clone GG101008Arch87 surface seawater of Puget Sound (JN592020.1)
Uncultured Nitrosopumilaceae archaeon clone GG101008Arch17 surface seawater of Puget Sound (JN592003.1)
Uncultured Nitrosopumilaceae archaeon clone GG101008Arch30 surface seawater of Puget Sound (JN591974.1)
Uncultured archaeon C1 R010 hydrothermal sediments in the Guaymas Basin (AF419636.1)
Nsub
Uncultured marine archaeal group 1 crenarchaeote clone ST-3K4A brine-seawater interface of the Shaban Deep of Red Sea (AJ347774.1)
Uncultured archaeon clone Ei 16SA 117 Brine-seawater interface of Erba Deep of Red Sea (KJ882001.1)
Uncultured archaeon clone Ki 16SA 124 Brine-seawater interface of Erba Deep of Red Sea (KJ882163.1)
Uncultured archaeon clone Ki 16SA 33 Brine-seawater interface of Erba Deep of Red Sea (KJ882072.1)
Uncultured archaeon clone Ei 16SA 119 brine-seawater interface of Atlantis II Deep brine pool (KJ882003.1)
Uncultured archaeon clone Ki 16SA 136 Brine-seawater interface of Kebrit Deep Red Sea (KJ882175.1)
Uncultured archaeon clone Ki 16SA 13 Brine-seawater interface of Kebrit Deep of Red Sea (KJ882052.1)
Uncultured archaeon clone Ki 16SA 121 Brine-seawater interface of Kebrit Deep Red Sea (KJ882160.1)
Uncultured archaeon clone Ei 16SA 131 Brine-seawater interface of Erba Deep of Red Sea(KJ882015.1)
Uncultured thaumarchaeote clone ErbaIntfcA RS14 Brine-seawater interface of Erba Deep brine pool (KF954269.1)
Uncultured archaeon clone Ei 16SA 10 Brine-seawater interface of Erba Deep of Red Sea (KJ881894.1)
Candidatus Nitrosopumilus sp. PS0 (KF957664.1)
Candidatus Nitrosopumilus adriaticus strain NF5 (CP011070.1)
KR737579.1 Nitrosopumilus sp. DDS1
Uncultured archaeon clone Ki 16SA 24Brine-seawater interface of Kebrit Deep of Red Sea (KJ882063.1)
Uncultured archaeon clone Ei 16SA 6 Brine-seawater interface of Erba Deep of Red Sea (KJ881890.1)
Uncultured archaeon clone Ei 16SA 47 Brine-seawater interface of Erba Deep of Red Sea (KJ881931.1)
Uncultured crenarchaeote clone sj14 marine sediment (FJ971125.1)
Uncultured crenarchaeote clone sj4 marine sediment (FJ971121.1)
Uncultured crenarchaeote clone sj13 marine sediment (FJ971124.1)
Candidatus Nitrosopumilus koreensis AR1 (CP003842.1)
Uncultured crenarchaeote clone ar11 marine sediment (FJ971114.1)
Uncultured archaeon clone Ni 16SA 53 Brine-seawater interface of Nereus Deep of Red Sea (KJ882252.1)
Uncultured crenarchaeote clone ar3 marine sediment (FJ971110.1)
Uncultured crenarchaeote clone ar1 marine sediment (FJ971108.1)
Candidatus Nitrosopumilus piranensis strain D3C (CP010868.1)
Candidatus Nitrosopumilus maritimus tropical seawater tank substratum at Seattle Aquarium (DQ085097.1)
Nitrosopumilus maritimus SCM1 strain SCM1 (NR 102913.1)
Uncultured thaumarchaeote clone AAA799 D11 (KF954226.1)
Uncultured thaumarchaeote clone AAA799 P11 brine-seawater interface of Atlantis II Deep brine pool (KF954230.1)
0.002

B

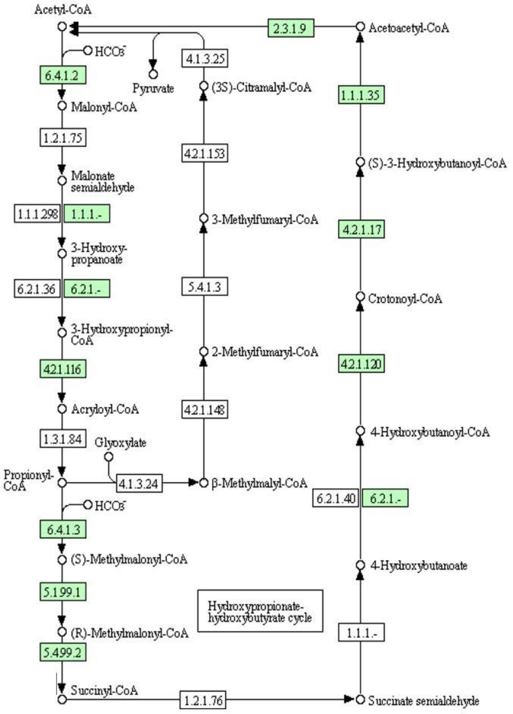

Acetyl-CoA
2.3.1.9
Acetoacetyl-CoA
6.4.1.2
Pyruvate
4.1.3.25
(3S)-Citramalyl-CoA
1.1.1.35
Malonyl-CoA
1.2.1.75
4.2.1.153
(S)-3-Hydroxybutanoyl-CoA
Malonate semialdehyde
1.1.1.298
1.1.1.-
3-Methylfumaryl-CoA
4.2.1.17
3-Hydroxy-propanoate
6.2.1.36
6.2.1.-
5.4.1.3
Crotonoyl-CoA
3-Hydroxypropionyl-CoA
4.2.1.116
2-Methylfumaryl-CoA
4.2.1.120
Acryloyl-CoA
4.2.1.148
4-Hydroxybutanoyl-CoA
1.3.1.84
Glyoxylate
Propionyl-CoA
4.1.3.24
β-Methylmalyl-CoA
6.2.1.40
6.4.1.3
(S)-Methylmalonyl-CoA
4-Hydroxybutanoate
5.1.99.1
Hydroxypropionate-hydroxybutyrate cycle
(R)-Methylmalonyl-CoA
1.1.1.-
5.4.99.2
Succinyl-CoA
1.2.1.76
Succinate semialdehyde
